# Comprehensive review of industrial wastewater treatment techniques

**DOI:** 10.1007/s11356-024-34584-0

**Published:** 2024-08-07

**Authors:** Shoma Kato, Yasuki Kansha

**Affiliations:** Organization for Programs on Environmental Sciences, Graduate School of Arts and Sciences, The University of Tokyo, 3-8-1 Komaba, Meguro-Ku, Tokyo 153-8902 Japan

**Keywords:** Wastewater treatment, Effluents, Industrial wastewater, Physical methods, Chemical methods, Biological methods, Water pollution, Advanced treatment

## Abstract

Water is an indispensable resource for human activity and the environment. Industrial activities generate vast quantities of wastewater that may be heavily polluted or contain toxic contaminants, posing environmental and public health challenges. Different industries generate wastewater with widely varying characteristics, such as the quantity generated, concentration, and pollutant type. It is essential to understand these characteristics to select available treatment techniques for implementation in wastewater treatment facilities to promote sustainable water usage. This review article provides an overview of wastewaters generated by various industries and commonly applied treatment techniques. The characteristics, advantages, and disadvantages of physical, chemical, and biological treatment methods are presented.

## Introduction

### Background of wastewater treatment

Water is an indispensable resource that sustains ecosystems, supports human life, and drives industry and agriculture. The demand for freshwater has been continuously growing with the global population. Of the water on Earth, 97% is saline and only 3% is freshwater (Oki and Kanae 2006). Only 0.5% of this freshwater is available for human use and exists in liquid form in rivers, lakes, ponds, and groundwater (Sangamnere et al. 2023). The severity of water scarcity has been underscored by the United Nations World Water Assessment Programme (WWAP); that is, the number of people residing in regions that experience water scarcity for at least 1 month annually is currently 3.6 billion and could increase to 4.8–5.7 billion by 2050 (WWAP 2018).

A comprehensive approach is needed to address growing water scarcity that includes water management and wastewater treatment and reuse (Bauer et al. 2020). The global increase in freshwater usage and wastewater generation has resulted in persistent water scarcity. The quantity of wastewater generated globally each year is estimated at 380 billion m^3^ and is expected to increase by 51% by 2050 (Qadir et al. 2020). The discharge of untreated wastewater remains a global issue. According to a WWAP report (2017), 80% of global wastewater is untreated and discharged, and Jones et al. (2021) have reported that 48% of global wastewater is untreated. The direct discharge of wastewater leads to the pollution of water bodies and groundwater, which can result in eutrophication (Qadir et al. 2020) and endanger the health of plants and animals (Ahmed et al. 2021a). Natural water bodies have self-purification ability, whereby some pollutants can be removed through natural physical, biological, and chemical means. However, the excess discharge of untreated wastewater surpasses the self-purification ability of natural water bodies (Pratiwi et al. 2023).

The implementation of wastewater treatment is a necessary but challenging task to prevent water pollution and meet water demand, especially in low-income regions. In high-income regions, such as North America, Western Europe, and Japan, 74% of the wastewater is treated, whereas 4% of wastewater is treated in low-income regions (Jones et al. 2021). High-income countries enforce wastewater quality regulations and possess the technology and infrastructure needed to install wastewater treatment plants (WWTPs), whereas low-income countries lack these resources. Consequently, those living in low-income regions may be exposed to wastewater and have limited access to clean water (WWAP 2017). Thus, global efforts are needed to increase the quantity of wastewater treated.

The properties of influent wastewater, such as the pollutant concentration, must be analyzed to determine WWTP specifications. Wastewater is largely classified by its generation source into municipal, agricultural, and industrial wastewater (Samer 2015). Industrial wastewater can be further classified into cooling, washing, and process wastewater (Crini and Lichtfouse 2019). Wastewater can be discharged from point and nonpoint sources. Point sources discharge wastewater from easily identifiable outlets, such as WWTPs of industrial facilities and municipal WWTPs. Nonpoint sources cannot be easily identified and include multiple sources, such as agricultural and urban runoff. Thus, point sources can be monitored and regulated more easily and are often more concentrated than nonpoint sources (Jones et al. 2021). Differences in water usage result in a variety of pollutants in wastewater that may contain toxins or pathogens harmful to human health and the ecosystem. Some common pollutants include organic compounds, inorganic compounds, phosphorus, nitrogen, and heavy metals (Akpor et al. 2014).

### Overview of wastewater treatment

**Fig. 1:**
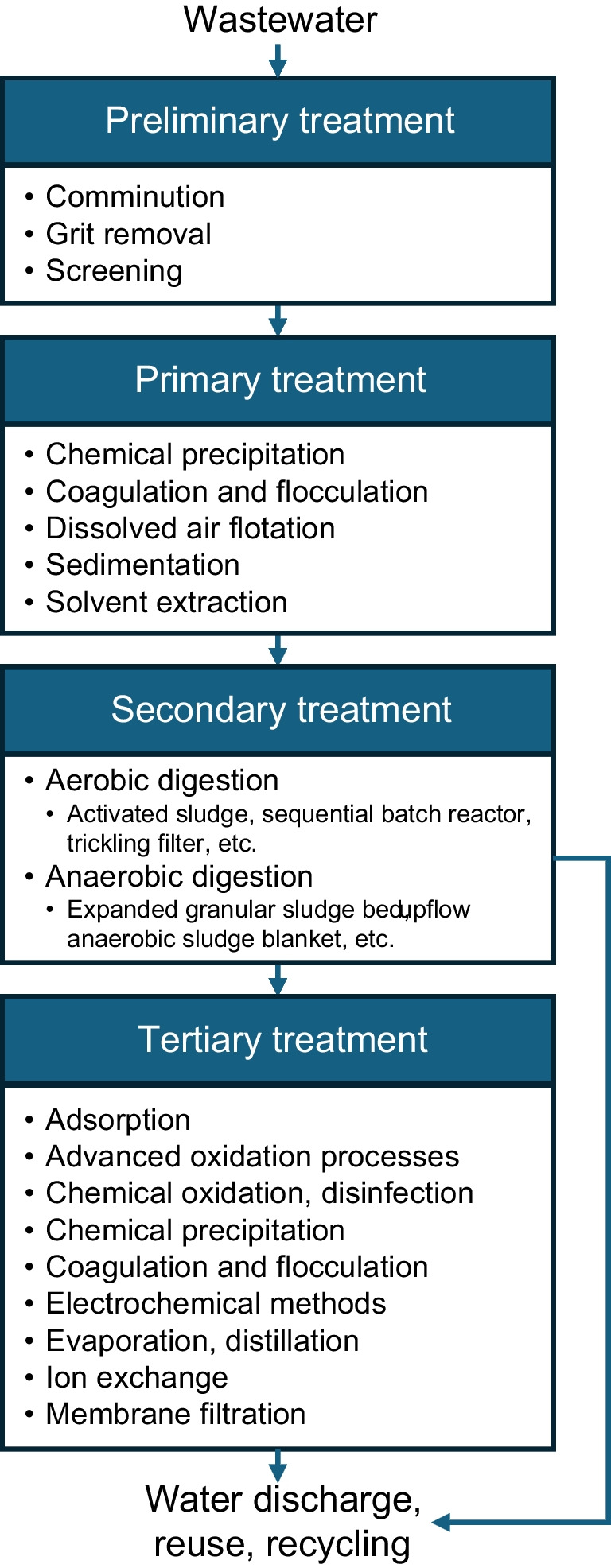
The general flow of a wastewater treatment process and the techniques used

Typically, WWTPs are designed to treat wastewater cost-effectively while achieving a desired water quality (Crini and Lichtfouse 2019). Wastewater treatment often requires the sequential use of various wastewater treatment techniques, each of which is suitable for removing certain contaminants. A wastewater treatment process generally consists of preliminary, primary, secondary, and tertiary stages (Naidoo and Olaniran 2013; Quach-Cu et al. 2018). The flow of a general wastewater treatment process and corresponding treatment techniques used are shown in Fig. 1.

Preliminary treatment removes large debris, grit, and solids from wastewater through processes such as screening, comminution, and grit removal. Next, primary treatment removes suspended solids, grit, fats, and oils through processes such as sedimentation and dissolved air flotation (DAF). Secondary treatment consists of using biological techniques to reduce organic matter, nitrogen, and phosphorus in wastewater through biochemical reactions such as conversion into biomass (Samer 2015). Tertiary treatment, also known as advanced treatment, is applied to further upgrade the treated wastewater to meet specific standards for water reuse or discharge. Tertiary treatment includes methods such as membrane filtration, adsorption, and chemical oxidation.

Treatment techniques can be classified into physical, biological, and chemical methods. Physical methods include screening, comminution, grit removal, sedimentation, DAF, adsorption, ion exchange, and membrane filtration. Biological treatment can be subdivided into aerobic and anaerobic treatment depending on oxygen availability. Chemical treatment includes precipitation, coagulation, flocculation, evaporation, distillation, membrane distillation, solvent extraction, electrochemical methods, chemical oxidation, and advanced oxidation processes (AOPs). The characteristics, advantages, and disadvantages of each method are detailed in “Treatment of industrial wastewater” section.

Industrial wastewater is often more toxic than municipal wastewater (Häder 2018). Industrial wastewater contains various contaminants at different concentrations depending on the industry (Ahmed et al. 2021a). Thus, industrial wastewater may not be adequately treated by municipal WWTPs, which are designed mainly for the removal of biochemical oxygen demand (BOD) (Abu Shmeis 2018). Effective treatment of industrial wastewater requires an analysis of the wastewater properties, such as the type and concentration of pollutants, and implementation of a suitable treatment process. Considering the variation in wastewaters across industries, WWTPs are implemented on a case-by-case basis using available treatment techniques. Treatment methods are selected by considering many factors, such as the influent wastewater characteristics, regulatory standards, land availability, technological availability, and economic viability.

### Objective of this review

The objective of this review paper is to examine the diverse types of contaminants found in wastewater generated across various industries and to evaluate the range of treatment techniques currently being implemented. An in-depth analysis is presented of the characteristics, advantages, and disadvantages of available physical, chemical, and biological wastewater treatment methods. Although numerous review papers have been written on singular treatments or specific industrial sectors, there is a limited number of holistic overviews covering the wide spectrum of wastewaters generated by multiple industries and corresponding treatment methods. This review bridges this gap by providing a comprehensive description of industrial wastewaters and the strengths and weaknesses of treatment technologies. This information is essential for selecting and integrating suitable treatment options that are cost-effective and sustainable as well as compliant with regulatory standards.

### Methods

The selection of industries for this study was based on the thorough review of academic literature and reports. The primary criteria for inclusion were the volume of wastewater produced, the diversity and complexity of pollutants, the environmental and health impacts, and regulatory focus.

The reference search was conducted across multiple academic databases, including Google Scholar, PubMed, ScienceDirect, and Web of Science, using keywords related to the specific industry, wastewater, and wastewater treatment. Additionally, the search for references on the wastewater treatment technologies was conducted by using the academic databases and consulting recent reviews in the field and tracing the citations. This methodology offers a detailed and balanced review, highlighting both the pollutants present in the wastewater of the selected industries and the technologies available for their treatment.

## Wastewater generated by various industries

Limited information is available on the overall volume of industrial wastewater generated and discharged globally. However, according to reports from the European Union (EU, which consists of developed countries), manufacturing generated the most wastewater among the industrial sectors (WWAP 2017). The major point sources of manufacturing industrial wastewater include industries such as petrochemicals, pharmaceuticals, pulp and paper, textiles, iron and steel metal manufacturing, and food.

The use of water for various purposes in industry generates wastewater with different characteristics, such as the types and concentrations of pollutants. Industrial wastewater may be heavily polluted and contain toxic pollutants. Industrial wastewater can be broadly categorized into cooling, washing, and process wastewater (Crini and Lichtfouse 2019). Wastewater generated from various industries and uses has diverse characteristics. The pollutants in industrial wastewater include solids, sediments, organic compounds, nutrients, and heavy metals. Considering the diversity and potential toxicity of industrial wastewater, a treatment technique that can sufficiently remove pollutants must be selected and implemented. The pollutants in wastewaters from the cement, chemical, food, iron and steel, pharmaceutical, pulp and paper, and textile industries are reviewed in this section. Wastewater treatment techniques used in each industry are summarized.

### Cement, concrete, and ceramics industry

Cement is an important material for constructing buildings and infrastructure, which drive economic development and urbanization. Table 1 shows the three main phases of cement production. Water is used for cooling and washing equipment and, if wet scrubbers are used, for removing particulate matter (PM). In some cases, water is used in the preparation of raw materials for clinker production (Perera et al. 2020). The used cooling water is typically recycled and reused in the process (Sharma et al. 2012). Cement production generates air pollutants, such as nitrogen oxides (NO_x_), particulate matter (PM), carbon dioxide (CO_2_), and sulfur dioxide (SO_2_) (Chen et al. 2015). Wet scrubbers are used to remove PM from the exhaust gas by trapping particles in water droplets, generating wastewater (Zhu et al. 2022). Other methods used to prevent air pollution include the use of simple membrane filters made of fabric, electrostatic precipitators, and bag filters (which are considered the best removal option for PM) (Zhu et al. 2022). Cement industry effluents contain suspended solids (such as calcium carbonate), dissolved solids (such as potassium hydroxide), sodium hydroxide, chlorides, and sulfates, BOD (approximately 5 mg/L), chemical oxygen demand (COD, approximately 60 mg/L), nutrients, and heavy metals (such as iron, zinc, and manganese (Meme and Nwadukwe 2016; Ipeaiyeda and Obaje 2017).

**Table 1:** Description of the cement production process (Teplická and Sedláková 2023)

Phase	Activity	Description
1. Mining	1. Quarry	Mining limestone and clay and sorting for white and gray cement production
	2. Crusher	Pre-crushing limestone to a size of 1 to 8 cm
	3. Conveyor	Transporting pre-crushed limestone from the quarry to the homogenization hall using belt conveyors
2. Processing raw materials	4. Homogenization	Mixing limestone with clay in the homogenization hall
	5. Grinding	Grinding the homogenized mixture to the required fineness to produce raw material flour
	6. Filter	Removal of dust from the exhaust gas
	7. Preheating	Preheating raw material flour before entering the rotary kiln
	8. Rotary kiln	Producing clinker by processing raw material flour at about 1450 °C, causing a series of chemical reactions
	9. Cooling of the clinker	Cooling the produced clinker to approx. 100 °C with air and storing it in silos. The heated cooling air is reused as combustion air in the furnace
3. Cement production, packing, and expedition	10. Completion of the process	Grinding clinker with mine slag, limestone, and gypsum to obtain cement
	11. Packing	Packing the cement into bags as per customer requirements
	12. Expedition	Shipping the cement via truck, train transport, etc

In concrete production, manufactured cement is mixed with water and aggregates (such as sand and gravel). The washing of truck mixers in a ready-mix concrete plant generates wastewater with a high pH that contains dissolved solids, cement, and other pollutants (Ekolu and Dawneerangen 2010). Concrete production is characterized by high water usage, where 150 L of freshwater is used to produce a cubic meter of concrete. Thus, the utilization of wastewater has been considered as a possible solution to reduce water consumption and wastewater discharge (Azeem et al. 2023).

Ceramics are a broad category of inorganic, nonmetallic materials that are typically hard, brittle, and heat-resistant. Ceramics are used in various applications for properties such as electrical and thermal insulation, corrosion resistance, and decorative appeal. Ceramic products include construction materials (such as tiles and bricks), refractories (such as crucibles and molds), pottery products, and toilets.

Ceramic manufacturing techniques span a wide range from hand-building to advanced industrial techniques. Although the specific manufacturing steps depend on the type of ceramic and the product, several key steps are common to all processes. First, the raw materials, such as clay, alumina, and silica, are prepared through processes such as mixing and grinding. Binders (colloids or polymers, such as polyvinyl alcohol and polyethylene glycol) and plasticizers (such as water, ethylene glycol, and stearic acid) are added during the forming of dry powders and plastics, whereas deflocculants, surfactants, and antifoaming agents are added during slurry processing (U.S. EPA 1996). Water is the most used liquid during these processes (U.S. EPA 1996). Next, the prepared raw materials are formed into desired shapes using various methods, such as pressing and casting. The formed ceramics are dried carefully and subjected to bisque firing to remove remaining water and impurities. A glaze is optionally applied to the bisque-fired ceramics. The glazed ceramic is fired again using a temperature, duration, pressure, and atmosphere appropriate for the type of clay and glaze. Effluents are generated during various steps of ceramics production, including glaze and slurry preparation, mold preparation, casting, and glazing (de Almeida et al. 2016). These effluents have high concentrations of total suspended solids (TSS) (2,000–10,000 mg/L) and dissolved solids (300–1,000 mg/L), moderate COD (500–1200 mg/L), and low concentrations of heavy metals (such as lead, cadmium, iron, copper, and manganese) (Dinçer and Kargı 2000). Effluent recycling is one way of avoiding effluent handling and reducing usage of water and raw materials (de Almeida et al. 2016).

#### Physical and chemical treatment

Wastewater from the cement industry has a high pH and turbidity and is treated by neutralization followed by sedimentation (Freeda Gnana Rani et al. 2005; Zhu et al. 2022). Wastewater from the concrete industry is similarly treated by neutralizing the pH and removing TSS. Coagulation and flocculation may be used to increase the settleability of TSS. De Paula et al. (2014) proposed a coagulation–flocculation treatment process using aluminum sulfate, *Moringa oleifera* powder, and floc sedimentation. The process achieved 90% turbidity removal, such that the treated water could be used to wash vehicles and flush toilets. Physical and chemical treatments of wastewater from the ceramic industry include adsorption, screening, sedimentation, filtration, coagulation, flocculation, and filtration (Pujiastuti et al. 2021). Pujiastuti et al. (2021) used a polyaluminum chloride coagulant to treat wastewater from the ceramic industry, achieving removals of up to 99.9% TSS, 98.23% COD, and 99.1% lead.

#### Biological treatment

Biological treatment is not commonly used for wastewater from the cement industry because the typical pollutants are TSS, dissolved solids, and heavy metals and the pH is high. However, some studies have been conducted on applying biological treatment to wastewater from the cement industry. Ali et al. (2021) investigated the use of a pilot-scale process consisting of a primary sedimentation tank, integrated fixed-film activated sludge (AS), and a final settling tank. The removals of TSS, COD, BOD, total nitrogen (TN), and total phosphorus (TP) were 94.5%, 87.8%, 90.8%, 75.9%, and 69.4%, respectively. The proposed treatment was found to be effective, satisfying the regulation standards in Egypt to reuse treated wastewater in agriculture. Biological treatment is not commonly used for wastewater from the ceramic industry. However, Dinçer and Kargı (2000) studied using AS to treat and reduce the BOD and COD of wastewater from the ceramic industry after chemical precipitation, pH adjustment, and nutrient balancing.

### Chemical industry

The chemical industry produces a broad range of chemicals, including commodity, specialty, and fine chemicals. These chemicals are used in various sectors, such as agriculture and manufacturing, to make final products. The basic chemicals produced by the petrochemical industry are particularly important because of subsequent use in the manufacturing of plastics, fibers, lubricants, and detergents. Petrochemicals are produced from fossil fuels, such as crude oil, coal, and natural gas or biomass (such as corn and sugarcane). The refining of crude oil provides fuels (such as liquefied petroleum gas, gasoline, and diesel) and chemical products (such as waxes, greases, asphalts, olefins, and aromatics) (Dincer and Zamfirescu 2014). The main petrochemical products are olefins (such as ethylene and propylene) and aromatics (such as benzene, toluene, and xylene) (Ren et al. 2009; Do et al. 2016).

Water usage in refineries for cooling, distillation, hydrotreating, and desalting generates wastewater (Ghimire and Wang 2019). Pollutants found in wastewater from the petrochemical industry include aromatics, hydrocarbons, sulfides, ammonia, and heavy metals (such as chromium, iron, nickel, and copper) (Radelyuk et al. 2019; Wake 2005; Yu et al. 2017). These compounds can be harmful to both the environment and human health. Petrochemical wastewater is treated by physical, biological, and chemical methods.

#### Physical treatment

Oily wastewater is commonly treated by using sedimentation and DAF to separate and remove oil (Abuhasel et al. 2021). Adsorption and ion exchange can be used to remove dissolved organics from petrochemical wastewater (Fakhru’l-Razi et al. 2009). Membrane separation can be used to remove oils, total organic carbon (TOC), and metal ions from oily wastewater (Yu et al. 2017). Some challenges encountered using membrane technology include cost, thermal stability, and corrosion resistance, which may be overcome by developing novel materials and combination with other treatment technologies (Yu et al. 2017).

#### Biological treatment

Aerobic and anaerobic treatment is commonly used to remove organics, ammonium, and sulfide from petrochemical wastewater because of advantages such as low cost and a high pollutant removal efficiency (Ghimire and Wang 2019). However, refractory organic compounds, such as aromatics, may be difficult to remove by traditional biological methods. Liu et al. (2014) found that wastewater from a petrochemical complex treated using AS still contained refractory organic compounds, such as alkanes, chloroalkanes, aromatics, and olefins. These compounds could be removed using a ponds-and-wetland system.

#### Chemical treatment

Coagulation, flocculation, and electrochemical technologies may be used to treat oily wastewater (Abuhasel et al. 2021). Advanced oxidation processes, such as heterogeneous photocatalysis, can be used to degrade refractory organic pollutants, such as phenolic compounds, in refinery wastewater (Diya’uddeen et al. 2011; Bustillo-Lecompte 2020).

### Food industry

The industrial manufacture of food is essential for human life and to meet global challenges, such as hunger and environmental sustainability. The food supply chain involves the production of crops and livestock, food processing, and logistics (Sun et al. 2017). Water is used for various processing purposes across different food industries. This water is categorized mainly into process water (such as that used as a raw material) and nonprocess water (such as that used for washing, cooling, and heating) (Abdel-Fatah 2023). Water usage requirements, such as the water quality and volume, vary across different sectors and processes in the food industry. Consequently, the generated wastewater can have varying characteristics, including the contamination level and volume. The generated wastewater may contain high levels of COD, BOD, TSS, TN, and TP resulting from various processes and nonprocess water usage. Most of the water used in many food industry sectors is employed for washing foodstuff and equipment, generating wastewater containing organics and nutrients.

The food industry can be categorized into several key sectors. These sectors can be ranked in terms of decreasing water consumption as meat, dairy products, other foods, fruits and vegetables, bakery products, grain mill and starch products, edible oils and fats, and fish and shellfish (Mark and Strange 1993; Ranken et al. 1997; Asgharnejad et al. 2021; Eurostat 2023). “Other foods” include sugar, coffee, tea, cocoa, chocolate, confectionery, condiments and seasonings, prepared meals, homogenized food, and dietetic food (Eurostat 2023). Table 2 summarizes the products, water usage, wastewater characteristics, and wastewater treatment methods for key sectors of the food industry. Sugar, coffee, and tea are included because of being important internationally traded products that require large quantities of water for production (Asgharnejad et al. 2021). In 2021, the estimated global production of green coffee, tea (i.e., green, black, and partly fermented tea), and raw cane or beet sugar was 10.50, 6.81, and 176.95 million tons, respectively (FAO 2024). Various physicochemical and biological technologies can be used to treat wastewater from the food industry. The treatment method is selected considering factors such as the wastewater characteristics, quality demand for the treated water, cost, and energy requirements. Technologies can be combined to improve the overall treatment efficiency. Considering the high water consumption and wastewater generation of the food industry, direct or indirect use of treated water and resource recovery, such as biogas production, is encouraged to mitigate water scarcity (Shrivastava et al. 2022).

**Table 2:** Summary of the products, water usage, and characteristics and treatment of wastewater for the food industry

Food industry sector	Products	Water uses	Wastewater pollution content	Wastewater treatment methods
Meat	Poultry, pork, beef, sheep meat, and meat products such as sausages and burgers (Kearney 2010; OECD/FAO 2021)	Most of the water is used for washing blood and debris from the meat (i.e. 44–60% for evisceration, 7–38% for offal washing, 9–20% for casing washing) (Fornarelli et al. 2017)	BOD, COD, TN, TP, and TSS such as blood, debris, meat, and bones. High total nitrogen from proteins in blood and debris (Bustillo-Lecompte and Mehrvar 2015; Philipp et al. 2021)	Physicochemical treatment (e.g. coagulation-flocculation, membrane, DAF, sedimentation, AOPs) and biological treatment (e.g. aerobic, anaerobic, upflow anaerobic sludge blanket (UASB), sequential batch reactor (SBR)) (Bustillo-Lecompte and Mehrvar 2015; Philipp et al. 2021)
Fish and shellfish	Chilled, frozen, salted, smoked, dried, and canned fish and shellfish products (Hall 1997)	Water is used for pre-processing and processing (e.g. heading, scaling, skinning filleting, cooking), storage, and transportation (Venugopal and Sasidharan 2021)	High salinity, BOD, COD, TSS, and nutrients from the seawater, blood, protein, guts, and flesh (Venugopal and Sasidharan 2021)	Physicochemical (e.g. sedimentation, adsorption, coagulation-flocculation, DAF, UF, RO, UV) and biological treatment (e.g. AS, aerobic lagoons, anaerobic,) (Venugopal and Sasidharan 2021)
Fruit and vegetables	Fresh, cold, frozen, dried, canned fruits and vegetables, processed products such as juice, jellies, and jams (Dauthy 1995)	About 88% of the water is used for washing and rinsing, and the rest is consumed for cleaning (Manzocco et al. 2015)	High BOD and COD content and low nitrogen and phosphorus content are contained in the effluent such as from washing (Puchlik and Struk-Sokołowska 2017; Puchlik 2018)	Physicochemical (e.g. sedimentation, ultrafiltration, screening, precipitation, chlorination, UV) and biological (e.g. aerobic, anaerobic, AS, constructed wetland) methods (Valta et al. 2017; Libutti et al. 2018; Puchlik 2018; Sehar and Nasser 2019)
Edible oils and fats	Edible oils such as olive, canola, palm, soybean, sunflower, rice bran, peanut, and coconut oil; edible fats such as butter, tallow, and lard (Teasdale et al. 2022)	Water is used for the pre-treatment of raw material, for the oil refining processes such as degumming, deodorization, and neutralization, for washing equipment, and for cooling and heating (Nweke et al. 2014; Ahmad et al. 2020)	High BOD, COD, TN, TP, TSS, TDS, sulfate, oil, and grease from pre-processing, and processing (Nweke et al. 2014; Ahmad et al. 2020). Phosphate and sulfate content results from soap usage for neutralization and the use of phosphoric acid for degumming (Ahmad et al. 2020)	Physicochemical treatment (e.g. coagulation-flocculation, membrane, adsorption, electrochemical, DAF, AOPs) and biological treatment (e.g. aerobic, anaerobic, UASB, microbial fuel cell) (Rajkumar et al. 2010; Nweke et al. 2014; Dkhissi et al. 2018; Sharma et al. 2018; Ahmad et al. 2020; Yaghmaeian et al. 2023)
Dairy	Fresh dairy products such as milk and yogurt and processed products such as butter, cheese, and milk powder (OECD/FAO 2021)	Extensive volume of water is used for washing and cleaning the equipment and facility (i.e. cleaning-in-place (CIP)) (Finnegan et al. 2018)	Dairy industry effluent, such as cheese production effluent, contains high COD, BOD, TN, carbohydrate, fat, and protein content (Bortoluzzi et al. 2017; Atasoy et al. 2020)	Physicochemical treatment methods (NF, RO, coagulation-flocculation, electrocoagulation, adsorption, AOPs) and biological (AS, aerated lagoon, UASB, algal, anaerobic SBR, membrane bioreactor), (Bortoluzzi et al. 2017; Atasoy et al. 2020; Asgharnejad et al. 2021; Kaur 2021)
Grain mill and starch	Grain seeds and flour of cereals such as corn, wheat, rice, and rye (Delcour et al. 2010)	Dry milling uses water for tempering and conditioning the grain seeds before grinding (Warechowska et al. 2016) Wet milling uses water for steeping and washing steps to separate starch, proteins, fiber, and lipids in the grain (Loubes et al. 2016; Ballester-Sánchez et al. 2019; Zhang et al. 2021)	High BOD, COD, TSS, TDS, oil and grease due to the washing of grains containing organic content such as proteins and starch (Asgharnejad et al. 2021; Zhang et al. 2021)	Physicochemical treatment (filtration, sedimentation coagulation-flocculation, ozone, UF) and biological treatment (algal, aerobic, anaerobic) (Asif and Khan 2016; Yi and Wang 2017; Ramachandran and Gangasalam 2019; Keerthana et al. 2020)
Bakery and farinaceous products	Bread, pastries, cookies, crackers, cakes, pasta, biscuits, and noodles (Hitzmann et al. 2022)	About half of the water consumed is used in the process, and the other half is used for cleaning and washing (Chen et al. 2005)	BOD, COD, TSS, TDS, TN, TP, oil, and grease due to the washing and cleaning effluent containing the used ingredients including oil, grease, flour, sugar, yeast, and detergent (Chen et al. 2005; Struk-Sokolowska and Tkaczuk 2018)	Physicochemical treatment (screening, coagulation-flocculation, sedimentation, DAF, electrochemical,) and biological treatment (AS, trickling filter, anaerobic treatment) (Chen et al. 2005; Struk-Sokolowska and Tkaczuk 2018; de Santana et al. 2018)
Sugar	Cane sugar, beet sugar (OECD/FAO 2021)	Water is used for washing raw ingredients, by-products, and equipment; cooling; heating; and extraction (Khakimova et al. 2022; Nouhou Moussa et al. 2023)	High BOD, COD, TSS content, and TN, TP, odors, and color (Prakash et al. 2015; Nouhou Moussa et al. 2023)	Physicochemical treatment (DAF, filtration, sedimentation, coagulation-flocculation, membrane, electrochemical, AOPs) and biological treatment (algal, aerobic, anaerobic filter, AS, UASB) (Fito et al. 2019; Sharma and Simsek 2020; Nouhou Moussa et al. 2023)
Coffee	Green coffee beans are obtained by wet or dry processing coffee fruit; roasted coffee beans (Murthy and Madhava Naidu 2012)	Water is used in wet coffee processing for sorting, pulping, fermentation, and washing to produce green coffee (Murthy and Madhava Naidu 2012; Ijanu et al. 2020; Alemayehu et al. 2020)	High BOD, COD, TSS, and TN content and color effluent generated by wet coffee processing (Dadi et al. 2018; Alemayehu et al. 2020)	Physicochemical treatment (e.g. electrochemical, membrane, adsorption, coagulation-flocculation, AOPs) and biological treatment (e.g. anaerobic, aerobic, AS, expanded granular sludge bed) (Rattan et al. 2015; Ijanu et al. 2020; Alemayehu et al. 2020)
Tea	Tea from the *Camellia sinensis* plant such as green tea, black tea, white tea, oolong tea, pu-erh tea, and cut-tear-curl (UNCTAD 2016)	Water is used for the boiler, domestic use, and cleaning equipment (Yadav and Kalaiyarasi 2015; Taulo and Sebitosi 2016)	High COD and BOD content and TSS, TDS, and color are contained in the effluent from washing the equipment (Yadav and Kalaiyarasi 2015; Taulo and Sebitosi 2016; Saha et al. 2019)	Physicochemical treatment (membrane, coagulation, photo-Fenton, sedimentation, sand filtration) and biological treatment (aerobic, constructed wetland, AS) (Sabaikai et al. 2014; Mwaka 2017; Saha et al. 2019)

### Iron and steel industry

Iron and steel are important drivers in modern society and economic development because of their use in infrastructure (such as roads and bridges, buildings, housing, machinery, and equipment in various industries), transportation (such as cars and trains), and consumer goods (such as tools and utensils). However, the production of iron and steel requires extensive usage of water and energy and generates wastewater containing toxic pollutants as well as CO_2_ emissions (Garg and Singh 2022).

The raw materials used to produce iron and steel include iron ore, coal for making coke, and limestone (Kumar et al. 2023). Iron and steel can also be produced from recycled scrap metal using electric arc furnaces (EAFs) (Yang et al. 2014). The steel industry uses a large quantity of water for operations, such as cooling, scrubbing, and descaling (Colla et al. 2017). Water consumption in a steel plant can range from 1 to 150 m^3^ per ton of steel depending on the location, plant configuration, and local regulations (Suvio et al. 2012). Either once-through cooling or recirculating cooling is used according to water availability, which depends on the plant location. Once-through cooling is used in coastal locations where seawater is abundant and accounts for approximately 80% on average of the water consumed in steel plants (Suvio et al. 2012).

Iron and steel manufacturing involves the processes described below (United States Environmental Protection Agency (EPA) 2008; Garg and Singh 2022).

1. Coke production: Metallurgical coke is produced by heating coal in an oxygen-free environment.
2. Sintering: Fine raw materials, including iron ore, limestone, and coke, are agglomerated at high temperatures. The product (which is called sinter) is fed to a blast furnace (BF).
3. Ironmaking: Materials containing iron, such as sinter, are reduced in the BF twice using hot gas. Alternatively, direct reduced iron can be produced from iron ore using natural gas, coal gas containing hydrogen, or CO, which does not require the production and use of coke.
4. Desulfurization of iron: Reagents, such as CaC_2_ and CaCO_3_, are injected into the molten iron. The reagents react with sulfur to produce slag, which is removed.
5. Steelmaking: Steel is produced in basic oxygen furnaces (BOFs), EAFs, or open hearth furnaces (OHFs). In a BOF, oxygen is injected into molten metal to remove impurities. In an EAF, current is run through scrap metal using carbon electrodes, and the scrap metal is melted and refined. An OHF is a shallow basin in which metal scrap and molten metal from the BF are heated, melted, and refined.
6. Product preparation: Processes, such as pouring the molten steel into ingots, reheating, casting, shaping, and rolling, are used to finish the product.

Water is used in most processes of steel production, including coke production, sintering, the BF and BOF stages, and rolling, for purposes such as cooling, quenching, and gas-cleaning (Suvio et al. 2012). In addition to these main steel manufacturing processes, a large quantity of water is used in supporting processes, such as power generation and equipment cooling (Suvio et al. 2012). These complex iron and steel production processes generate wastewater containing a large variety of pollutants. The main pollutants in wastewater from the steel industry include COD, NH_3_–N, volatile phenols, cyanide, TSS, heavy metals, and petroleum (Tong et al. 2018; Choudhury et al. 2023). The largest water consumption occurs in ironmaking and steelmaking, whereas most of the pollution in iron and steel industry wastewater results from coking (Tong et al. 2018).

Reducing the water intake and wastewater discharge of the iron and steel industry requires the implementation of wastewater treatment and water recycling or reuse. Wastewater from the iron and steel industry is treated by physical, biological, and chemical methods. A suitable wastewater treatment method can be selected based on the wastewater pollutants, concentration, quantity, and characteristics, which vary across plants and processes, such as coking, ironmaking, and steelmaking (Lawal and Anaun 2022). Although conventional primary, secondary, and tertiary treatments are used in industry, emerging processes, such as hybrid biological processes, AOPs, and membrane filtration, have been effectively used for pollutant removal (Rawat et al. 2023).

#### Physical treatment

Adsorption is a simple, low-cost, and effective method for removing a variety of pollutants, including heavy metals in wastewater (such as that from the iron and steel industry) (Feng et al. 2022b). Activated carbon is widely used as an adsorbent because of its effectiveness but can be relatively costly (De Gisi et al. 2016). The iron- and steelmaking industry is unique in that byproducts, such as metallurgical slag, can be used as low-cost alternative adsorbents (Manchisi et al. 2020). Nguyen et al. (2018) reported that the byproducts of coal fly ash and blast furnace slag can be used as low-cost adsorbents to effectively remove heavy metals, such as Pb, Cu, Cd, Cr, and Zn. Unmodified raw coal fly ash has been demonstrated as an adsorbent for treating coking wastewater (Wang et al. 2018). The raw coal fly ash achieved 90% COD removal and could be regenerated by the Fenton process.

Membrane filtration is sufficiently effective for treating wastewater from the iron and steel industry that the water can be reused at the industrial scale. However, membrane fouling is a major problem that decreases the permeate flux and increases energy usage (Liang et al. 2023; Lin et al. 2023). The presence of salts in wastewater can cause problems, such as membrane fouling, and recycled water containing salt can cause salt deposition or corrosion of equipment (Colla et al. 2016). Lin et al. (2023) reported that deposits of Fe and Mn ions and oxides in integrated steelwork wastewater may cause fouling of ultrafiltration (UF) membranes and should be removed before UF. Liang et al. (2023) suggested that further investigation of reverse osmosis (RO) membranes is needed to prevent fouling because these membranes are used for recycling wastewater from the iron and steel industry. Huang et al. (2011) reported that constructed wetlands are an effective pretreatment before UF and RO for reducing iron and manganese concentrations in wastewater from the iron and steel industry and to improve the quality of the treated water for reuse. The constructed wetland, UF, and RO system achieved 98% desalination. An RO system can effectively reduce the concentration of salts, electrical conductivity, and total dissolved solids (TDS), enabling the treated water to be reused and increasing the equipment lifespan (Colla et al. 2016).

#### Biological treatment

Aerobic and anaerobic treatment, such as AS, is used to treat wastewater from the iron and steel industry. Considering that the complexity and toxicity of this wastewater may reduce the performance of conventional biological treatment, hybrid biological processes, such as anoxic–oxic–anoxic–oxic (AOAO), anaerobic–anoxic–oxic (AAO), and anaerobic–anoxic–oxic–oxic (AAOO), have been developed for the efficient removal of pollutants (Rawat et al. 2023). Biological treatment plants can use various microorganisms (e.g., bacteria, algae, yeast, and fungi) that have different metabolic pathways to remove organic and inorganic pollutants in wastewater from the iron and steel industry (Kajla et al. 2021). Hybrid biological processes employing diverse microbial communities have been used at the industrial scale to meet effluent standards. Ma et al. (2015) collected sludge from coking WWTPs in China that employ different processes, including anaerobic–oxic (AO), AAO, anaerobic–oxic–oxic (AOO), AAOO, and AOAO. An analysis of the composition of the microbial community in the sludge showed that most sludge contained *Thiobacillus, Comamonas, Thauera, Azoarcus,* and *Rhodoplanes*. The key parameters for the biological treatment, such as the operation mode, flow rate, and temperature, were found to affect the makeup of the microbial community and thereby, the pollutant removal performance.

#### Chemical treatment

Chemical methods, such as coagulation–flocculation, AOPs, and electrochemical techniques, have been used to treat wastewater from the iron and steel industry (Garg and Singh 2022). Coagulation–flocculation has been used in conventional integrated wastewater treatment systems as a primary treatment to remove pollutants, such as oils and heavy metals (Das et al. 2018). Coagulation–flocculation can also be used as a pretreatment to prevent filtration membranes from being fouled by effluent (Lin et al. 2023). Coking wastewater may be treated by AOPs, such as ozonation (Wang et al. 2019), catalytic ozonation (Feng et al. 2022a), Fenton oxidation (Chu et al. 2012; Kwarciak-Kozłowska and Włodarczyk 2020), photolysis (Włodarczyk-Makuła et al. 2016), and photocatalysis (Sharma and Philip 2016), to degrade pollutants and increase the biodegradability of the effluent in subsequent biological treatment. Studies have been performed on using electrochemical methods, such as electrocoagulation and electrochemical oxidation, to treat coking wastewater. Wang et al. (2022) developed Ti/SnO_2_RuO_2_–Yb electrodes for the electrochemical oxidation of coking wastewater, achieving 85.06% COD removal and 60.59% TOC removal. Ozyonar and Karagozoglu (2015) studied how pretreated coking wastewater was affected by electrocoagulation and electrochemical peroxidation using a direct pulse current. Electrochemical peroxidation was found to be more effective than electrochemical oxidation in removing COD, TOC, phenol, CN^–^, and SCN^–^. Mierzwiński et al. (2021) investigated the electrocoagulation of coking wastewater using floc characterization, mathematical modeling, and designing an industrial-scale electrocoagulation reactor. Overall, chemical treatment methods can be integrated with physical or biological methods to improve pollutant removal and promote wastewater reuse in the iron and steel industry. Emerging chemical methods are effective but present challenges, such as a high cost and difficulty in scale-up for industrial use.

### Pharmaceutical industry

Pharmaceutical products are crucial to society for the prevention, treatment, and management of various medical conditions, enhancing overall public health and well-being. A large quantity of water is used as a raw material, ingredient, and solvent in the industrial production of pharmaceuticals and separation processes, such as extraction and washing (Gadipelly et al. 2014). Chemical synthesis and fermentation processes are the major processes that generate wastewater during pharmaceutical production (Gadipelly et al. 2014). Pharmaceuticals used in households and agriculture can appear in wastewater. The variety of pharmaceutical products, intermediates, and raw materials results in a diversity of wastewater contaminants. Wastewater from the pharmaceutical industry contains organics that may not be biodegraded, such as antibiotics, anti-inflammatories, steroids, hormones, antidepressants, and spent solvents (Rana et al. 2017; Samal et al. 2022). Discharge of and exposure to pharmaceuticals may have detrimental effects on plants and animals (Gadipelly et al. 2014; Samal et al. 2022).

#### Physical treatment

Membrane technologies, such as those based on polymer membranes, are low-energy simple strategies for removing pharmaceutical active compounds from wastewater. However, membrane fouling is an important limitation and has been mitigated by membrane modification (Ratnasari 2023). The use of activated carbon for the removal of pharmaceuticals has been studied, but further research needs to be conducted on the cost-effectiveness and application of this technology to real wastewater (Rasras et al. 2021).

#### Biological treatment

Aerobic and anaerobic biological treatments, such as AS, membrane bioreactors (MBRs), moving bed biofilm reactors (MBBRs), and constructed wetlands, may be used to remove pharmaceuticals from wastewater (Moghaddam et al. 2023). The conventional activated sludge (CAS) treatment often used in municipal WWTPs cannot sufficiently remove persistent micropollutants. However, MBRs are a promising technique for removing these micropollutants because the longer sludge retention time and higher sludge concentration used results in a higher efficiency (Tiwari et al. 2017).

#### Chemical treatment

Electrochemical coagulation has been studied at the lab scale for the removal of pharmaceuticals from wastewater, but further research needs to be carried out on this technology using real wastewater and performing a cost analysis (Alam et al. 2021). Refractory pharmaceuticals can be effectively degraded using AOPs, such as ozone (O_3_), hydrogen peroxide (H_2_O_2_), Fenton oxidation, and photocatalysis, which use highly reactive radical species (Gadipelly et al. 2014).

### Pulp and paper industry

Pulp and paper products, such as newspapers, books, packaging materials, and tissue products, play a vital role in society. Wastewater is generated by the pulp and paper industry through processes such as wood preparation, pulp manufacturing, pulp bleaching, and papermaking (Ashrafi et al. 2015). The use of different processes and raw materials results in diverse wastewater characteristics, such as the quantity generated and the pollutant concentration. The quantity of generated wastewater can be as high as 60 m^3^/ton of paper produced (Thompson et al. 2001). Preparing wood to produce chips includes harvesting, debarking, chipping, and screening (Amândio et al. 2022). The generated wastewater contains TSS, BOD, dirt, grit, and fibers (Pokhrel and Viraraghavan 2004). Wood pulp can be manufactured from the prepared wood by mechanical, chemical, or hybrid methods (Toczyłowska-Mamińska 2017). Mechanical pulp production involves grinding or refining wood. In hybrid processes, wood pulp is produced by cooking with alkali and milling (Nong et al. 2020). Four common chemical pulping processes are kraft, sulfite, neutral sulfite semichemical, and soda (Cheremisinoff and Rosenfeld 2010). The kraft process accounts for 90% of chemical pulp production because of a high product quality and low production cost (Argyropoulos et al. 2023). During this process, an alkaline solution of sodium hydroxide and sodium sulfide, also known as white liquor, is used to dissolve lignin from cellulose fibers under high temperature and pressure. The dilute spent liquor is concentrated using evaporators to approximately 60%–65% solids (Young et al. 2003; Cheremisinoff and Rosenfeld 2010). The resulting “black liquor” contains organics, such as lignin and hemicellulose, and inorganics, such as salts and sulfur compounds, which are generated as byproducts (Valderrama et al. 2021). The black liquor is burned for energy and to recover chemicals (Young et al. 2003). The wood pulp is then bleached with compounds containing chlorine or oxygen (Bajpai 2018), which generates bleaching wastewater with a high COD and TSS as well as low biodegradability (Eskelinen et al. 2010) and refractory organic compounds, such as adsorbable organic halides (AOX) (Patel et al. 2021). In the final papermaking process, additives, such as dyes, may be used to make colored paper, whereby the generated wastewater may contain particulate waste as well as organic and inorganic compounds, such as the dyes used (Patel et al. 2021). Wastewater generated from the pulp and paper industry is treated physically, biologically, or chemically to reduce water pollution and recover energy and materials.

#### Physical treatment

Sedimentation and flotation are used to remove TSS in wastewater from pulp and paper mills. Primary clarifiers can effectively remove more than 80% TSS (Thompson et al. 2001). Another commonly used technique, DAF, can remove 80%–98% TSS (Miranda et al. 2009). Manago et al. (2018) investigated the removal of fibers using DAF with polyaluminum chloride as a coagulant, where more than 81.7% TSS was removed. Filtration using membranes of various pore sizes can be used to remove pollutants in wastewater from the pulp and paper industry, such as organics, ions, and AOX (Esmaeeli et al. 2023). Membranes used for wastewater treatment in the pulp and paper industry must be able to withstand extreme conditions, such as high temperatures and pHs, as well as antifouling measures for organic and inorganic foulants (Esmaeeli et al. 2023). Valderrama et al. (2021) developed nanofiltration (NF) membranes to treat black liquor. These membranes have a high rejection of organics with a TOC removal of 92.5% and high salt removals, such as 88.7% sulfate, 73.21% Na^+^, and 99.99% Mg^2+^. Adsorption methods, such as the use of activated carbon and zeolite, have been investigated for the treatment of wastewater from the pulp and paper industry by removing heavy metals, such as Cd, Ba, and Cu (Aprianti et al. 2018), as well as COD and color (Kapatel et al. 2022).

#### Biological treatment

In the pulp and paper industry, aerobic and anaerobic treatment is used to reduce the high organic content of wastewater. Aerobic treatment, such as AS, aerated lagoons, and stabilization ponds, have traditionally been used, whereas anaerobic treatment, such as the upflow anaerobic sludge blanket (UASB) reactor, is promising because of advantages such as lower sludge generation, lower energy consumption, and biogas generation (Patel et al. 2021; Esmaeeli et al. 2023). Aerobic treatment is more suitable for low- and medium-strength effluents, whereas anaerobic treatment is better for treating high-strength effluents (Buyukkamaci and Koken 2010; Esmaeeli et al. 2023). Buyukkamaci and Koken (2010) performed a cost analysis of 96 treatment plants with 12 flow schemes, including physical, chemical, and biological treatment, and reported that biological processes are most economical for treating wastewater from the pulp and paper industry.

#### Chemical treatment

Chemical methods for treating wastewater from the pulp and industry wastewater include chemical precipitation, coagulation and flocculation, and AOPs, such as ozonation, ozone/H_2_O_2_, photocatalysis, and Fenton oxidation (Ashrafi et al. 2015; Esmaeeli et al. 2023). Kaur et al. (2020) reported that using a conventional alum coagulant and chitosan flocculant to treat wastewater from pulp and paper mills removed 81% TSS and 78% COD. Eskelinen et al. (2010) compared the removals of COD from simulated wastewater containing model compounds of wood extractives (abietic acid, linoleic acid, and β-sitosterol) using various chemical treatment methods, including ultrasonic (US) irradiation combined with Fenton-like oxidation (Fe^3+^/H_2_O_2_), photo-Fenton degradation (Fe^3+^/H_2_O_2_/UV), chemical precipitation using CaO, and electrooxidation. Although the highest COD removal was obtained using chemical precipitation with CaO, combination with subsequent biological treatment is needed to meet the legislative COD limit of 200 mg/L. Ribeiro et al. (2020) reported maximum AOX removals of 85% and 95% by using the Fenton and photo-Fenton processes, respectively, to treat bleaching wastewater from a kraft pulp mill over a 10-min period. Studies were performed on increasing the COD removal efficiency by combining biological treatment with AOPs, such as O_3_, O_3_/UV, UV, UV/H_2_O_2_, heterogeneous photocatalysis (TiO_2_/UV and ZnO/UV), Fenton oxidation, and photo-Fenton oxidation. Specifically, O_3_ treatment has been implemented at an industrial scale in pulp and paper mills (Hermosilla et al. 2014). Combining AOPs with biological treatment may improve the wastewater treatment efficiency and reduce costs (Hermosilla et al. 2014).

### Textile industry

The textile industry provides essential goods to society, such as clothing and fabrics. Fabric is manufactured by successive processing of raw materials (cellulose, protein, and synthetic fibers) to yarn to greige fabric to the fabric product.

Fabric production involves dry and wet processes. The wet processes require a large quantity of water and generate highly polluted wastewater (Yaseen and Scholz 2019). Water consumption can range from 30 to 150 L/kg of cloth according to the type of fiber being processed, with the effluent containing 200–600 mg/L BOD, 1,000–1,600 mg/L total solids, and 30–50 mg/L TSS (Azanaw et al. 2022). Cotton is the main raw material used for fabric production, accounting for 60% of the earnings of the industry (Velusamy et al. 2021). Wet processing of cotton includes sizing, desizing, scouring, bleaching, mercerization, dyeing, printing, and finishing (Holkar et al. 2016). Wet processes generate wastewater with varying characteristics resulting from the differences in the raw materials and process used. Table 3 shows the characteristics of wastewaters from different wet processes (Correia et al. 1994; Kant 2012; Sarayu and Sandhya 2012; Holkar et al. 2016; Azanaw et al. 2022).

**Table 3:** Characteristics of wastewaters generated from wet fabric production processes (Correia et al. 1994; Kant 2012; Sarayu and Sandhya 2012; Holkar et al. 2016; Azanaw et al. 2022)

Process	Description	Characteristics of the wastewater
Sizing	Starch, polyvinyl alcohol (PVA), carboxymethyl cellulose (CMC), etc. are used to increase strength and reduce friction to produce fabric from yarn. Equipment washing results in wastewater	A small amount of high BOD, COD, and TSS wastewater
Desizing	The sizing material is removed by solubilization, hydrolysis, or oxidation. Desizing methods include the use of enzymes, oxidative agents, acids, hot water, and detergent	High BOD for starch sizing and enzyme desizing, lower BOD for synthetic sizing (PVA, CMC, etc.) removal Desizing agents are also present in the effluent
Scouring	Substances such as waxes, oils, herbicides, etc. are removed using hot alkali such as caustic soda with detergent and soap	High BOD. Effluent contains low biodegradable or toxic pollutants such as herbicides, pesticides, and waxes
Bleaching	To remove the natural color of the fiber and make it white, bleach such as hydrogen peroxide, peracetic acid, sodium hypochlorite, or sodium chlorite is used	Low to moderate BOD, but high TSS. Bleach such as H_2_O_2_ and chloride remain
Mercerization	The cotton fabric is treated with concentrated caustic soda NaOH (18–24% by weight) to increase the luster and dye affinity, followed by acid wash for neutralization	Low BOD and TSS. Highly alkaline, and NaOH remains
Dyeing and printing	Synthetic or natural dyes are used for coloring the fabric. Thick dye paste is used for printing onto the fabric. The effluent is complex due to the different dye types and the process	Low TSS, but moderate to high TDS, moderate BOD. Heavy metals, salt, and residual dyes can be toxic
Finishing	Finishing processes are applied to improve the fabric properties, such as waterproofing, softening, and UV protection	Low volume, but BOD, TSS, and toxins such as chlorophenols may be present

Wastewater from the dyeing and printing process is diverse because of the numerous dyes used and variations in the dyeing process. Dyes can be classified according to their chemical structure (such as azo, nitro, anthraquinone, cyanine, and carbonyl) or the type of fiber being dyed (cellulose, protein, and synthetic) and application (such as direct, acid, basic, cationic, direct, reactive, and mordant) (Correia et al. 1994; Mustroph 2014). Synthetic dyes have been widely used for their color range, brightness of color, and fastness (Kant 2012). Azo dyes are the most commonly used synthetic dyes because of a low production cost, color variety, and fastness (Piaskowski et al. 2018; Al-Tohamy et al. 2022), accounting for 60%–70% of the synthetic dye industry (Slama et al. 2021). Untreated dye wastewater is harmful to aquatic and terrestrial life as well as the human skin, liver, nervous system, kidney, and reproductive system. (Al-Tohamy et al. 2022). Untreated dye effluents block the transmission of sunlight in water bodies and inhibit photosynthesis, leading to oxygen depletion and low biodegradability by aerobic microorganisms and disruption of the aquatic ecosystem (Slama et al. 2021). Heavy metals, such as lead, chromium, cadmium, and copper, as well as metals may appear in wastewater because of the use of metal-complex dyes (Khan et al. 2022). These heavy metals are toxic and harmful to aquatic life and human health, causing health problems, such as cancer and cardiovascular disease (Khan et al. 2022). To prevent these problems and meet effluent standards, textile wastewater must be treated before being released into the environment or reused. Physical, biological, and chemical methods are used to treat textile wastewater.

#### Physical treatment

Physical treatment methods, such as adsorption, membrane filtration, and ion exchange, can remove 85%–99% of dyes from effluent (Samsami et al. 2020). Adsorption using materials such as clay, zeolite, and activated carbon has been proposed as an efficient and low-cost method for the removal of heavy metals and dyes from wastewater (Velusamy et al. 2021). Membrane processes, such as microfiltration (MF), UF, nanofiltration (NF), and RO, for the removal of dyes, salts, and other auxiliary chemicals from textile wastewater have been investigated mostly in lab-scale studies and some pilot and full-scale studies, where over 95% removals of COD, turbidity, and color have been attained (Keskin et al. 2021). In particular, good performance has been reported using hybrid systems, such as AS followed by UF and RO (Keskin et al. 2021). Ion exchange using synthetic and natural resins for dye removal has been studied at the lab-scale but has a high cost and limited applicability to dyes (Khan et al. 2023; Singh et al. 2022).

#### Biological treatment

Biological treatment of textile effluents includes aerobic, anaerobic, and combined processes using bacteria, fungi, and algae (Holkar et al. 2016; Bhatia et al. 2017). A wide variety of dyes can be degraded by using a combination of microorganisms that are compatible with and capable of degrading dyes and their intermediates. Biological treatment is low-cost, eco-friendly, and generates low quantities of sludge compared to other methods (Bhatia et al. 2017; Samsami et al. 2020). Generally, anaerobic treatment can be used to treat high-COD effluents and remove color, whereas aerobic treatment can be used to treat low-COD effluents. Rongrong et al. (2011) developed a lab-scale hybrid anaerobic baffled reactor for treating desizing effluents containing polyvinyl alcohol (PVA), achieving 42.0% COD removal while collecting methane-containing biogas, which can be used for other purposes. In the industrial treatment of textile wastewater, aerobic and anaerobic AS or MBRs are combined with technologies such as coagulation–flocculation, RO, and ozonation (Paździor et al. 2019). Compared to AS, MBRs can achieve higher biomass loadings by using supports on which biofilms can grow, and the presence of various microorganism species enables efficient removal of dyes, COD, BOD, TSS, phosphorus, and heavy metals (You et al. 2007).

#### Chemical treatment

Coagulation–flocculation has been used in the textile industry as a cost-effective method for color removal from textile wastewater despite excessive sludge generation (Verma et al. 2012). Coagulation–flocculation may be used after secondary biological treatment and before tertiary membrane filtration to prevent fouling by removing colloids, TSS, and color (Aragaw and Bogale 2023). Studies have been performed on using AOPs, such as ozone-based processes, H_2_O_2_, photocatalysis, and Fenton oxidation, to remove refractory pollutants from textile wastewater (Paździor et al. 2019). Bilińska et al. (2016) performed a comparative analysis on the removal of Reactive Black 5 using O_3_, UV/O_3_, O_3_/H_2_O_2_, O_3_/UV/H_2_O_2_, and H_2_O_2_/UV. The ozone-based processes—O_3_ and O_3_/H_2_O_2_—were cost-effective and could be used as a pretreatment before biological treatment to remove color and improve biodegradability. Heterogeneous photocatalysis has been investigated for the treatment of dye wastewater using semiconductor photocatalysts (TiO_2_ and ZnO, in particular) for their good photocatalytic activity and availability (Donkadokula et al. 2020).

## Treatment of industrial wastewater

Industrial wastewater is treated using physical, biological, and chemical techniques. Each treatment method has advantages and disadvantages regarding factors such as the efficacy of removing specific pollutants, the treatment volume, ease of use, cost, energy usage, and chemical consumption. In many cases, these technologies are combined to achieve efficient removal of multiple types of pollutants, while reducing the total treatment cost.

### Physical methods

Physical methods involve the removal of contaminants by exploiting physical and mechanical properties for separation (Pirzadeh 2022). These methods include screening, comminution, grit removal, sedimentation, DAF, adsorption, ion exchange, membrane filtration, evaporation, distillation, and membrane distillation.

#### Screening, comminution, and grit removal

Screening, comminution, and grit removal are used as pretreatment methods before primary clarification. The main purpose of these methods is to protect downstream equipment and improve the effectiveness of subsequent treatment stages by removing or grinding large solids and debris.

During screening, large solids and debris are removed by passing wastewater through a series of screens or mesh filters to prevent damage to downstream pipes and equipment. Various types of screens, including coarse and fine, are used according to the size and characteristics of the solids and debris to be removed. Coarse screens, such as bar screens, usually have openings with sizes of 6-mm or larger (U.S. EPA 2003). Fine screens usually have openings of between 1.5 and 6 mm in size (U.S. EPA 2003). To remove finer solids, very fine screens with openings of between 0.2 and 5 mm can be used, and microscreens with openings of between 0.001 and 0.3 mm can be used to further treat the secondary effluent, which may still contain fine solids (Prabu et al. 2011). Typically, coarse screens are used near the inlet to capture large solids, followed by using fine screens to capture small particles. The debris captured on the screens is removed manually or mechanically and disposed of in landfills, incinerated, or ground and returned to the wastewater stream (Prabu et al. 2011). The collected solids typically contain various materials, such as plastic, paper, rags, food, and feces from human activity (Szostkova et al. 2012). Screening is used in the textile industry to remove large solids (such as yarn, lint, fibers, and rags) (Azanaw et al. 2022) and in the meat processing industry to remove bones and meat debris (Philipp et al. 2021).

Comminution can be used as an alternative to screening to reduce the size of solids in wastewater by grinding or shredding (Deluise et al. 2005). Comminutors consist of a screen and a rotating drum with slots and cutting teeth to shred solid materials that accumulate on the screen (McLeary 2004). Comminution itself does not remove solids from wastewater. The crushed solids are removed in a subsequent grit chamber and sedimentation tank (Ahmed et al. 2021b). Considering that the ground particles are not removed and can damage the downstream equipment, comminutors are not commonly installed in newer WWTPs (McLeary 2004).

Grit chambers are used to remove heavy particles, such as sand and gravel, from wastewater to prevent damage to downstream equipment, such as pumps and pipes. There are several types of grit-chamber configurations, such as aerated, vortex, and horizontal flows as well as hydrocyclones (U.S. EPA 2003). To remove solids efficiently, the type of grit chamber used is determined by many factors, such as the particle characteristics, settling velocity, space availability, maintenance requirements, energy consumption, and cost. The variable particle density warrants direct measurement of the settling velocity (Plana et al. 2020).

#### Sedimentation

Sedimentation is a simple and common method for removing TSS from wastewater, which reduces BOD and COD (Jover-Smet et al. 2017). Primary sedimentation tanks, also known as primary settling tanks and primary clarifiers, are placed after screening and grit removal and before secondary biological treatment in conventional WWTPs. In sedimentation tanks, the wastewater velocity is reduced to cause TSS, organic matter, and other particles to settle to the bottom of the tank and form a layer of sludge. The clarified effluent is collected near the top of the tank around the wastewater surface.

Sedimentation tanks can be rectangular or circular. Rectangular tanks have a lower construction cost and can have a longer retention time but are less effective for treating wastewater with high TSS, whereas circular tanks have a lower maintenance cost and easier sludge collection but a shorter retention time. Short-circuiting is more likely to occur in circular tanks than in rectangular tanks (Hirom and Devi 2022). The efficiency of sedimentation tanks is determined by many parameters, such as the particle characteristics, settling velocity, tank dimensions, and wastewater and flow characteristics (Ferdowsi et al. 2022). Considering the complexity of the settling characteristics of suspended particles, experimental data have been used to develop empirical models for sedimentation tanks (Christoulas 1998; Martínez-González et al. 2009; Jover-Smet et al. 2017). As a result of advances in computational technology, computational fluid dynamics (CFD) has recently been used to model, design, and simulate sedimentation tanks, which has improved our understanding of tank hydrodynamics and thereby, tank design (Hirom and Devi 2022). The sedimented solids are mechanically removed by equipment, such as sludge scrapers and sludge pumps.

Secondary sedimentation tanks are also used in conventional treatment plants after secondary biological treatment, such as AS. The functions of secondary sedimentation tanks are settling sludge containing microorganisms to produce a clear effluent and thickening sludge for subsequent recirculation and storage (Patziger et al. 2012). Secondary sedimentation tanks have also been designed using empirical models (Gao and Stenstrom 2018). Computational fluid dynamics models have also been developed for secondary sedimentation tanks. These models have provided insights for tank design, such as the effect of the structure and position of the inlet on turbulence (de Almeida et al. 2020; Gao and Stenstrom 2018).

Sedimentation effectively clarifies wastewater by removing TSS and BOD_5_ at ratios of 50%–70% and 25%–40%, respectively (Jover-Smet et al. 2017). Sedimentation tanks can have complex hydrodynamics but are relatively low-cost and simple to design and operate. However, the disadvantages of these tanks, such as long retention times for settling fine particles, may lead to large tank volumes. Very fine particles and dissolved content are difficult to remove using sedimentation tanks.

#### Dissolved air flotation

Dissolved air flotation is used to clarify wastewater, where small air bubbles are used to remove TSS, BOD, COD, oils, and grease. Bubbles are generated by dissolving and saturating air under pressure into water and releasing the air into a flotation tank. The generated air bubbles attach to particles, which consequently float to the surface. The floating particles are removed by a skimming device and disposed. Flocculants and coagulants, such as polymers, ferric chloride, and aluminum sulfate, are often added to the wastewater to aggregate suspended particles, oils, and grease (Musa and Idrus 2021). Dissolved air flotation is used to treat many industrial wastewaters, such as those produced by the pulp and paper (Miranda et al. 2009), petrochemical (Yu et al. 2017), mineral processing (Rajapakse et al. 2022), and food industries (Shrivastava et al. 2022). For example, DAF can remove 70%–80% BOD and 30%–90% COD from slaughterhouse wastewater (Musa and Idrus 2021). For papermill wastewaters, 80%–90% TSS removal can be achieved by removing particles, such as fines, fillers, and ink (Miranda et al. 2009). The effectiveness of DAF depends on factors such as the bubble size distribution, gas–liquid mass transfer, hydrodynamics, wastewater characteristics, and tank geometry (Rajapakse et al. 2022).

Dissolved air flotation offers advantages over sedimentation, such as a shorter retention time, smaller space requirements, and faster removal of small and low-density particles (Rodrigues and Rubio 2007; Crini and Lichtfouse 2019). However, there are challenges associated with DAF, such as high operation and maintenance costs resulting from the energy requirements and maintenance costs for equipment (Yu et al. 2017; Musa and Idrus 2021). The addition of coagulants and flocculants may incur additional costs for DAF.

#### Ion exchange

The ion-exchange process consists of using a resin to remove dissolved ions and pollutants from water through exchange with similarly charged ions. Ion-exchange resins are solid materials made of a polymer matrix with functional groups attached by covalent bonds (Carolin et al. 2017). Some common polymer matrices include polystyrene, polyacrylic, phenolic, and polyalkylamine resins (de Dardel and Arden 2008). The resins are porous and have a large specific surface area for effective ion exchange. Conventional ion-exchange resins are bead-shaped with typical diameters between 0.04 and more than 1 mm (Fink 2013).

Ion-exchange resins can be largely categorized into cationic and anionic types. Cation-exchange resins are commonly used in softening applications to replace magnesium and calcium ions with sodium ions (Samer 2015). At the industrial scale, wastewater is passed through a column packed with an ion-exchange resin. The saturated resin can be regenerated by flushing the column with a sodium solution (Samer 2015). Anion-exchange resins can be used to exchange negatively charged ions, such as nitrate, sulfate, chloride, and bicarbonate (Gomaa et al. 2021). Anion-exchange resins can be regenerated by treatment with a basic solution, such as a sodium hydroxide solution or an ammonium hydroxide solution. Cationic and anionic resins can be classified by their functional groups, including the strongly acidic cation (SAC) with a sulfonic group, weakly acidic cation (WAC) with a carboxylic group, strongly basic anion (SBA) with a quaternary ammonium group, and weakly basic anion (WBA) with primary, secondary, or tertiary amino groups, and chelating with functional groups, such as iminodiacetic and phosphinic groups (Chen et al. 2021). The resin is selected depending on criteria such as the target ions to be removed, the wastewater characteristics, economic feasibility, and the desired water quality.

Ion exchange has been used for numerous purposes, such as water softening, demineralization, deionization, deacidification, removal of impurities (such as nitrates and heavy metals), decoloring, separation, and dehydration (de Dardel and Arden 2008). Thus, ion exchange is used in many fields, such as agriculture, food processing, chemical synthesis, laboratory use, wastewater treatment, and hydrometallurgy (Ijanu et al. 2020). Studies have been performed on using ion exchange for industrial wastewater treatment, such as to treat textile-dyeing wastewater (Khan et al. 2023) and remove fluoride (Wan et al. 2021), phenol (Anku et al. 2017), and heavy metals (Barakat 2011). Ion exchange has been shown to be an effective wastewater treatment but is not widely used industrially because of drawbacks, such as high associated costs and the limited selectivity of conventional resins (Crini and Lichtfouse 2019; Khan et al. 2023).

#### Adsorption

Generally, adsorption refers to the change in concentration of a substance relative to those of neighboring phases at the interface between two phases, including liquid–gas, liquid–liquid, solid–liquid, and solid–gas interfaces (Dąbrowski 2001). Solid adsorbents are used to remove organic and inorganic pollutants from wastewater. There are two types of adsorption: physisorption and chemisorption. Physisorption is the adhesion of an adsorbate onto an adsorbent surface through van der Waals forces. Chemisorption involves the formation of covalent or ionic bonds between an adsorbate and an adsorbent. Physisorption is weak, nonspecific, and reversible, whereas chemisorption is strong, more specific, and often irreversible. Many factors affect the effectiveness of adsorption, such as the temperature, pH, use of stirring, contact time, adsorbent dosage, and initial concentration (Sukmana et al. 2021; Chai et al. 2021).

Adsorbents used in water treatment include natural and synthetic materials, such as activated carbon, clay, biosorbents, graphene oxide, and various nanomaterials. (Tran 2023). Among various available adsorbents, activated carbon is the most popular and is widely used for wastewater treatment because of advantages such as a high specific surface area, wide applicability to many pollutants, and regeneration ability (De Gisi et al. 2016). However, activated carbon is expensive and can be costly to regenerate (Chai et al. 2021). The nonselectivity of activated carbon must be traded off against its wide applicability. Studies have been performed on using low-cost adsorbents, such as agricultural wastes (e.g., orange peel, banana peel, and rice husks) and industrial wastes (e.g., flue ash, red mud and bagasse ash) (Rashid et al. 2021). Li et al. (2019) reviewed the key requirements and analyzed the feasibility of using bioadsorption for industrial-scale treatment of dye wastewater. Bioadsorption was determined to be a competitive technology if the adsorbent used has a good adsorption/desorption performance and is reused numerous times.

Adsorption can be implemented at various stages of industrial wastewater treatment depending on the target pollutant, concentration, and desired treated effluent quality. For example, adsorption can be combined with technologies such as ozone, DAF, and coagulation to treat papermill wastewater and remove toxic pollutants, color, and COD (Thompson et al. 2001). Adsorption may also be used for secondary or tertiary treatment of wastewater containing oil and grease, such as those from petroleum refineries and metalworks (Pintor et al. 2016).

#### Membrane filtration

Wastewater can be filtered through semipermeable membranes with various pore sizes to separate pollutants, such as colloids, microorganisms, organics, salts, and ions. Semipermeable membranes are selectively permeated by substances with particular sizes and charges (Breite et al. 2019). Wastewater can be treated using MF, UF, NF, and RO membranes (Keskin et al. 2021). Asymmetric membranes are used in a pressure-driven process for their high flux and mechanical stability (Strathmann 2005). The membranes can be composed of polymers (such as polyethylene, polytetrafluorethylene, and polypropylene) or inorganic materials (such as ceramics, zeolites, and silica) (Ezugbe and Rathilal 2020).

Microfiltration membranes have relatively large pore sizes of 0.1 μm or more and are often used to pretreat wastewater before UF, NF, and RO (Behroozi and Ataabadi 2021). These membranes can separate larger particles, such as TSS, colloids, and organic matter at relatively low operating pressures of 0.02 to 0.5 MPa (Zioui et al. 2023). Ultrafiltration membranes have pore sizes of 0.001 to 1 μm and are used to remove pollutants, such as TSS, organics, oils, and pigments (Ezugbe and Rathilal 2020). Nanofiltration membranes have pore sizes of 1 to 5 nm and can therefore separate pollutants with relatively low molecular weights and reject pollutants such as sugar, salt, minerals, heavy metals, oils, and dyes (Mulyanti and Susanto 2018). Reverse osmosis membranes have pore sizes of a few angstroms and can separate sodium and chloride ions, making RO a promising technology for seawater desalination (Jamaly et al. 2014). Reverse osmosis membranes have small pore sizes that enable removal of all pollutants but are susceptible to fouling, must be operated under high pressure, and are more expensive than other membranes (Nqombolo et al. 2018).

Membrane filtration is a versatile technology for industrial wastewater treatment and can be combined with other technologies to achieve sufficiently high water quality for reuse. For example, membranes have been used in combination with biological treatment in MBRs. Membrane filtration can be used to treat wastewater from many industries, including pulp and paper (Valderrama et al. 2021), textiles (Keskin et al. 2021), electroplating, and petroleum (Barakat 2011). Some advantages of membrane filtration are the production of high-quality treated water, a smaller footprint than that of conventional filtration, and the ability to be installed in existing WWTPs. Some challenges associated with membrane filtration are higher overall costs compared with those of conventional WWTPs and susceptibility to fouling (Othman et al. 2022).

#### Evaporation, distillation, and membrane distillation

Evaporation, distillation, and membrane distillation are thermal wastewater treatment processes that can separate and concentrate pollutants in wastewater. The evaporated wastewater may be collected by condensation.

Evaporation ponds are shallow, open-air basins that are lined with materials, such as clay and synthetic materials, to prevent wastewater seepage. Water is evaporated by solar irradiation, resulting in the concentration of contaminants, precipitation of crystalline salts, and sediment accumulation. The accumulated solids are removed regularly and disposed. Evaporation ponds have various applications, such as the treatment of oil-produced water and mine wastewater and the rejection of brine from desalination plants and other industrial wastewater (Izady et al. 2020). Advantages of evaporation ponds include straightforward application and low capital and operational costs. Some disadvantages of evaporation ponds are the environmental and health impact of the release of heavy metals, pesticides, VOCs, CO_2_, and CH_4_ (Amoatey et al. 2021). Evaporation ponds require large land usage and solar irradiation and are therefore suitable for dry and warm locations with low-cost land (Abdeljalil et al. 2022).

Other evaporation techniques consist of using equipment such as flash evaporators, condensers, and distillation columns. Wastewater is converted to water vapor using heating devices, such as heat pumps and heating elements. The water vapor is then condensed and collected as distilled water. A portion of the wastewater may be left behind that contains unevaporated salts and other solids. Evaporation techniques can be used to treat a wide variety of wastewaters and are especially useful for desalination. However, steam generation has a large energy demand. Organics that evaporate at low temperatures may enter the treated water stream. To lower the energy demand for heating and removal of organics, Yang et al. (2018) proposed a desalination system with a low-temperature heat pump. A COD removal of 97% was achieved at 48 °C, producing 3 kg of treated water in 1 h for a power consumption of 250 W. Evaporation technologies have been developed to reduce energy consumption and improve the separation efficiency. Among evaporation technologies, multieffect evaporation is used most often because of maturity and high efficiency (Lu et al. 2017). Multieffect evaporation is implemented using a series of single-effect evaporators, where the vapor generated from one evaporator is used to heat the next evaporator to reduce energy consumption. Mechanical vapor recompression is an alternative emerging technology in which generated vapor is compressed and reused to heat the feed. Mechanical vapor recompression is mainly used for desalination and has advantages such as a higher energy efficiency than multieffect evaporation, compactness, and low-temperature operation (Liang et al. 2013). Some advantages of evaporation technologies are a high recovery rate, recoverability of both high-quality water and salts, wide applicability, and no use of supplementary materials. Some disadvantages of evaporation technologies are high capital costs, high energy consumption, and complexity (Mizuno et al. 2013, 2015; Lu et al. 2017).

Membrane distillation is a thermal separation process in which a hydrophobic porous membrane is used to pass water vapor while rejecting pollutants. Membrane distillation can be divided into four types depending on the permeation side: direct contact in which both sides of the membrane surface contact vapor, air-gap in which the permeation side has an air gap, sweeping gas in which a cold inert gas is used to transfer vapor from the permeate side, and vacuum in which a vacuum is applied to the permeate side by a pump at a lower pressure than the saturation pressure of the volatile molecules (Yan et al. 2021). Membrane distillation has been considered one of the most promising technologies for the treatment of saline wastewater and can also be used to treat oily wastewater by being combined with other processes to reduce fouling (Kalla 2021). Some advantages of membrane distillation are low working pressures (to prevent fouling), high selectivity, and low sensitivity to the feed solute concentration. Some disadvantages of membrane distillation are a lower throughput than RO, pore-wetting risk, and a high energy demand (Shirazi and Dumée 2022).

### Chemical methods

Chemical treatment involves the use of chemicals, such as inorganics (iron and aluminum salts) and organics (cationic, anionic, and nonionic polymers). Some examples of chemical treatment methods are coagulation–flocculation and chemical precipitation (for increasing the settleability of pollutants), chemical oxidation, and AOPs (for degradation of organics, pH adjustment, and disinfection). Chemical treatment is often combined with other biological and physical treatment processes as a pretreatment or post-treatment strategy.

#### Chemical precipitation

Chemical precipitation removes dissolved pollutants from wastewater as solid particles. This technique is effective for the removal of heavy metals and is widely used in industry because of low costs and facile operation (Yadav et al. 2019). Counterions are added to reduce the solubility of dissolved ions, which are then removed through precipitate formation (Zueva 2018). These precipitates must be separated out using methods such as sedimentation and filtration. Flocculants may be used to improve the settleability of precipitants (Ojovan and Lee 2014). Chemical precipitation has the advantages of low capital costs and simple operation but also has disadvantages, such as the operating costs of using chemical precipitants and sludge disposal (Wang et al. 2005).

Dissolved metals can be precipitated as hydroxides, sulfides, and carbonates. The most widely used method for hydroxide precipitation involves the addition of alkaline agents, such as calcium hydroxide (lime) or sodium hydroxide (Dahman 2017). Lime is the most cost-effective alkaline agent for wastewater treatment (Zueva 2018). The addition of lime results in the formation of metal hydroxides and calcium ions:

1 $${M}^{n+}+Ca{\left(OH\right)}_{2}\leftrightharpoons M{\left(OH\right)}_{n}+C{a}^{2+}$$

The optimal pH for hydroxide precipitation depends on the dissolved metal. Considering the amphoteric nature of metal hydroxides, decreasing or increasing the pH may cause precipitates to resolubilize. Thus, it is challenging to treat wastewater containing different metals because multiple steps are required to remove the metal precipitates at their optimal pHs.

Sulfide precipitation involves the addition of sulfide ions usually generated from H_2_S, Na_2_S, CaS, (NH_4_)_2_S, or NaHS (Estay et al. 2021). The sulfide ions react with metals to form metal sulfide precipitates:

2 $${M}^{n+}+{S}^{2-}\leftrightharpoons {M}_{n}S$$

Sulfide precipitation offers advantages over hydroxide precipitation, such as less soluble precipitates, faster reaction rates, and better settling, as well as disadvantages, such as the sensitivity of the reaction system to the sulfide dosage and problems associated with the usage of excess sulfide (Lewis 2010).

Carbonate precipitation is an alternate method involving the use of sodium carbonate or calcium carbonate. Sodium carbonate participates in the following reactions, producing a metal carbonate precipitate and CO_2_ that can attach to and float the precipitate (Zueva 2018):

3 $${M}^{n+}+nNaC{O}_{3}\leftrightharpoons nMC{O}_{3}+nN{a}^{+}$$

4 $$nMC{O}_{3}+{H}_{2}O\leftrightharpoons C{O}_{2}+{\left(MOH\right)}_{n}C{O}_{3}$$

Quiton et al. (2022) compared the efficacies of carbonate and hydroxide precipitation for the removal of cobalt and copper from electroplating wastewater. Compared to hydroxide precipitation, carbonate precipitation achieved a higher removal efficiency of both metals at a lower pH of approximately 7–8, but generated a larger sludge volume.

#### Coagulation and flocculation

Unlike chemical precipitation, coagulation and flocculation remove TSS and colloids without a phase change. Coagulation and flocculation often occur simultaneously but are different treatment processes. Coagulation refers to the destabilization of a suspension or solution, whereas flocculation refers to the agglomeration of destabilized particles into large flocs (Bratby 2016). The generated flocs are separated by sedimentation, filtration, or air flotation.

Mixing is an important factor that affects the overall process performance. Coagulation and flocculation have different optimal mixing speeds and times. Rapid mixing is employed for coagulation, whereas slow mixing is employed for flocculation (Saritha et al. 2017). Yu et al. (2011) investigated the effect of rapid and slow mixing on coagulation and flocculation, using aluminum sulfate hydrate (alum) as a coagulant and kaolin clay as a model suspension. Increasing the time for rapid mixing decreased the final floc size, whereas increasing the speed of slow mixing decreased the floc size. Rapid mixing can cause floc breakage because of high shear and change in the floc surface properties, which affects the coagulation efficiency. Other important factors that affect the coagulation and flocculation efficiencies include the pH, coagulant and flocculant dosage, temperature, and the presence of anions (such as bicarbonate or sulfate) (Ersoy et al. 2009).

Conventional chemical coagulants include alum, ferric sulfate, ferric chloride, and polyaluminum chloride, to which natural coagulants and flocculants derived from animals, plants, and microorganism have been considered as alternatives (Badawi et al. 2023). Inorganic coagulants and flocculants have disadvantages, such as pH sensitivity, sludge generation, and leaching of metal ions from sludge to groundwater, which has resulted in increasing use of polymer flocculants that can form large flocs at low dosages (Maćczak et al. 2020). Commonly used flocculants include nonionic flocculants (such as polyacrylamide), cationic flocculants (such as polydiallyldimethylammonium chloride), and anionic flocculants (such as copolymers of acrylamide and ammonium) (Dao et al. 2016).

The advantages of coagulation and flocculation include simplicity, effective removal of colloids and suspended particles, and effective settling of sludge. The disadvantages of these processes include high operating costs incurred by continuous addition of coagulants and flocculants, large sludge generation, and sludge disposal costs (Iwuozor 2019).

#### Solvent extraction

Solvent extraction can be used to remove and recover valuable materials from wastewater. This technique has been used commercially for materials recovery, such as in the petroleum, wool, and pharmaceutical industries (Lo and Baird 2003). The first step in solvent extraction is contacting wastewater with an immiscible solvent. The solvent selectively extracts the target compound, known as the solute, from the wastewater. Sufficient contact results in a solute-rich solvent (the extract) and a solute-depleted effluent (the raffinate). The extract and raffinate are separated, the solute is recovered from the extract, and the solvent is recycled. The raffinate is the treated wastewater, which can be further treated, discharged, or recycled depending on the demanded water quality. Contact between the solvent and the wastewater and solute extraction are achieved using various extractors, such as mixer–settlers, agitated columns, and packed columns, whereas solvent regeneration and solute recovery can be accomplished by distillation and gas stripping (Chang 2020).

Solvent extraction is widely used for the treatment and recovery of wastewater with high concentrations of phenolic compounds, such as wastewater from coal gasification (Feng et al. 2017). Yang et al. (2006) developed a solvent extraction process using methyl isobutyl ketoneas the solvent to treat wastewater from coal gasification containing 5,000 mg/L phenol. In a trial plant with a wastewater flow of 2 t/h, 93% of phenols were recovered. The recovered phenols provided economic benefits that could compensate for the operational cost of the process. The advantages of using solvent extraction to treat wastewater include selectivity for specific pollutants, recovery of valuable materials, solvent regeneration, and no sludge generation (Chang 2020). The disadvantages of this process include the investment costs associated with the use of specialized equipment and solvents and the potential environmental and health impact of solvent use.

#### Electrochemical methods

Electrochemical technologies involve the application of electricity through electrodes. The most studied processes include electrocoagulation (EC), electroflotation (EF), electrochemical oxidation (EO), electroreduction (ER), and electrodialysis (ED) (Sillanpää and Shestakova 2017).

##### Electrochemical coagulation and electrochemical flotation

In EC and EF, an electric current is applied to an anode and cathode in a reactor to produce destabilization agents, such as Al and Fe, and gas bubbles (Emamjomeh and Sivakumar 2009). Under an applied electric current, metal cations are generated at the anode and hydroxide ions and hydrogen gas are produced at the cathode. In EC, metal hydroxides are generated by the combination of metal cations and hydroxide cations. The metal hydroxides neutralize charged contaminants, and the neutralized contaminants are adsorbed by sweep coagulation, resulting in the formation of flocs (Das et al. 2022). In EF, the flocs are separated by hydrogen gas bubbles that adhere to the flocs and float the flocs to the surface or by sedimentation (Emamjomeh and Sivakumar 2009).

Similar to conventional coagulation–flocculation, EC can be used to remove contaminants from wastewater, such as TSS, TOC, oils, heavy metals, COD, color, and turbidity (Das et al. 2022). The use of EC has been investigated to treat wastewater from the dairy, textile, petroleum, pulp and paper, and pharmaceutical industries (Boinpally et al. 2023). The advantages of EC include the effective removal of colloids, no addition of chemicals, low sludge generation, and simple operation. Electrocoagulation also has disadvantages, such as periodic replacement of the sacrificial anode, electrode fouling, the possibility of metal hydroxide dissolution, and power consumption (Sivaranjani et al. 2020; Boinpally et al. 2023).

##### Electrochemical oxidation

Electrochemical oxidation involves both direct and indirect oxidation. During direct oxidation, pollutants adsorb onto the anode surface and an electron is directly transferred between the anode and the pollutant. During indirect oxidation, reactive species, such as reactive oxidation species and chlorine active species, are generated at the electrode surface and react with pollutants (Garcia-Segura et al. 2018). Garcia-Segura et al. (2018) investigated using EO to treat wastewaters, such as those from the petroleum, pulp and paper, and pharmaceutical industries, by removing COD, TSS, and recalcitrant organics. Electrodes, especially the anode where oxidation occurs, are key EO components that affect the cost and efficiency of the process. Thus, effort has been expended in developing anode materials with high performance, low cost, and high stability (Qiao and Xiong 2021). The advantages of EO include the ability to degrade recalcitrant pollutants, a small footprint, no addition of chemicals, and reduced secondary pollution. The disadvantages of EO are high energy consumption, high cost of some electrodes (such as those based on noble metals and diamond), challenges with mass producing some electrodes, possible corrosion and fouling of electrodes, and electrode replacement.

##### Electrochemical reduction

Electrochemical reduction is an emerging technology that involves either direct or indirect reduction. During direct reduction, electron transfer occurs between the cathode and the pollutant adsorbed on the cathode. During indirect reduction, the cathode reduces a mediator, and the reduced mediator reduces the pollutant (Mousset and Doudrick 2020). Electrochemical reduction has been studied for the detoxification and conversion of toxic organics into value-added materials, denitrification, removal and recovery of metals (Xue et al. 2023), and decolorization (Sala and Gutiérrez-Bouzán 2012). The advantages of ER are no chemical addition, a low footprint, metal removal and recovery, and the conversion of pollutants into value-added materials. The disadvantages of ER include the high cost of noble-metal electrodes, high energy consumption, the need to control competition with the hydrogen evolution reaction, and the possibility of some corrosion. Compared to EO, ER has been the subject of fewer studies, cannot mineralize pollutants, and has slower kinetics (Xue et al. 2023).

##### Electrodialysis

Electrodialysis involves the application of an electric field and ion-exchange membranes t(IEMs) to separate ions, such as dissolved salts. Electrode compartments contacting the anode and cathode are placed on the outer sides of ED units. Between the electrodes, there are alternating layers of anion exchange membranes (AEMs) and cation exchange membranes (CEMs) separated by spacers. Under an applied electrical potential, cations migrate toward the cathode by passing through the CEMs and anions migrate toward the anode by passing through the AEMs. Cations are blocked by the AEMs, and anions are blocked by the CEMs, resulting in compartments with alternating concentrated and dilute solutions (Mohammadi et al. 2021). The number of cell pairs in an ED stack depends on the scale of the units, ranging from a few cell pairs for lab-scale units to several hundreds of pairs for pilot-scale units (Mohammadi et al. 2021). The IEMs are selective and separate molecules based on their charge.

Electrodialysis has been studied to treat industrial wastewater containing salts, such as those from oil and gas extraction, the petrochemical and coal mining industries, and power plants (Gurreri et al. 2020). The use of ED has been considered for the recovery of metals, such as those from the metal deposition and electroplating industries (Arana Juve et al. 2022). The advantages of ED include high salt removal, metal removal, low susceptibility to scaling, no addition of chemicals, and low operating pressures. The disadvantages of ED are high capital costs and energy demand, membrane fouling, and the inability to remove nonionic pollutants (Zhao et al. 2019; Mir and Bicer 2021; Arana Juve et al. 2022).

#### Chemical oxidation

Conventional chemical oxidation involves using various oxidizing agents to degrade organic pollutants in wastewater and disinfect biologically treated wastewater. Various oxidizing agents have been used, such as permanganate, O_3_, H_2_O_2_, chlorine, and persulfate (Devi et al. 2016). Chlorination has been commonly used to treat wastewater before discharge or reuse. Rodríguez‐Chueca et al. (2015) compared the efficacy of using chlorination to disinfect *Escherichia coli* in municipal wastewater with that of using NaClO and various AOPs, including UV, H_2_O_2_/solar irradiation, and photo-Fenton oxidation. Although the optimal disinfection/cost ratio was obtained using chlorination, chlorine can generate carcinogenic halogenated byproducts that pose health and environmental risks. H_2_O_2_ is another oxidizing agent that has been used to reduce BOD, COD, and odors to further improve the quality of wastewater treated by physical or biological treatment (Ksibi 2006). Doltade et al. (2022) used O_3_ and H_2_O_2_ as oxidizing agents to treat wastewater from the polymer industry, achieving COD reductions of 85% and 91%, respectively, from an initial COD of approximately 1920 ppm and demonstrated the synergistic effect of combining the two oxidants.

The advantages of chemical oxidation are versatility and effectiveness in removing a wide range of organic contaminants, a relatively short contact time for treatment compared to biological methods, odor and color removal, and disinfection. The disadvantages of chemical oxidation are the environmental impact of the oxidizing agent used and intermediates produced, operation costs associated with the production, storage, transportation, and usage of oxidants, and the need to use pretreatment.

#### Advanced oxidation processes

Advanced oxidation processes are emerging water treatment methods that can degrade trace toxic organics in wastewater and upgrade treated wastewater for reuse. Reactive species, such as hydroxyl radicals (HO•), are generated and nonselectively oxidize organic pollutants in wastewater to nontoxic substances, such as CO_2_ and H_2_O. These methods can effectively treat wastewater containing recalcitrant organics that are difficult to remove by conventional treatment, such as pharmaceuticals, pesticides, phenols, and dyes. As a result of growing interest in water reuse and the need to meet stricter water pollution regulations, AOPs are increasingly being used to upgrade effluents by removing persistent pollutants. Advanced oxidation processes include O_3_/H_2_O_2_/UV, Fenton oxidation, photocatalysis, and sonolysis.

##### O_3_, H_2_O_2_, and UV

The strong oxidants—O_3_ and H_2_O_2_—can be used alone or in various combinations with each other and UV, such as O_3_/UV, H_2_O_2_/UV, O_3_/H_2_O_2_, and O_3_/H_2_O_2_/UV. During ozonation, organics can be degraded by direct oxidation (reaction with O_3_) or by indirect oxidation (reaction with hydroxyl radicals) (Chiang et al. 2006). The combined use of O_3_, H_2_O_2_, and UV has been proposed to improve the organic removal efficiency (Matsumoto et al. 2021). These methods, especially O_3_/H_2_O_2_/UV, have been used to treat wastewater from the textile, pharmaceutical, petroleum, and aquaculture industries by efficiently removing emerging pollutants (Angeles Amaro-Soriano et al. 2021). The advantages of these technologies are nonselective oxidation of pollutants by radical species, complete mineralization, no generation of halogenated byproducts, disinfection, simple operation, and the ability to upgrade treated wastewater for reuse. The disadvantages are the capital and operating costs of ozone generation, UV irradiation, and H_2_O_2_ usage as well as the risks posed by handling O_3_ and H_2_O_2_.

##### Fenton oxidation

During Fenton oxidation, HO• radicals are generated by using H_2_O_2_ and iron ions as a homogeneous catalyst under acidic and ambient conditions. The generally accepted mechanism is given below (Bautista et al. 2008):

5 $$F{e}^{2+}+{H}_{2}{O}_{2}\to F{e}^{3+}+HO\cdot +H{O}^{-}$$

6 $$F{e}^{3+}+{H}_{2}{O}_{2}\to F{e}^{2+}+H{O}_{2}\cdot +{H}^{+}$$

Photo-Fenton oxidation, which combines Fenton oxidation with UV–vis irradiation, has been used to improve organic degradation. The generation of HO• is realized by the decomposition of H_2_O_2_ under light irradiation and the regeneration of Fe^2+^ through the following reactions:

7 $$F{e}^{3+}+{H}_{2}O+h\nu \to F{e}^{2+}+HO\cdot +{H}^{+}$$

8 $$F{e}^{3+}+{H}_{2}{O}_{2}+h\nu \to F{e}^{2+}+H{O}_{2}\cdot +{H}^{+}$$

Fenton and photo-Fenton oxidation have been investigated to treat wastewater from industries such as oil, textile, and pulp and paper (Machado et al. 2023). The advantages of Fenton oxidation include simplicity, effective pollutant degradation, availability of Fe^2+^ and H_2_O_2_, and environmental safety. The disadvantages of this method include the need for sludge disposal, pH control, and high chemical inputs (Bello et al. 2019).

##### Photocatalysis

Heterogeneous photocatalysis involves the use of a semiconductor photocatalyst that is activated by light irradiation. The photocatalyst absorbs photons with sufficient energy to generate electron–hole pairs that participate in reactions. Organic pollutants may be degraded directly on a photocatalyst surface or indirectly by generated HO• (Oturan and Aaron 2014).

In photocatalytic applications, TiO_2_ has been widely used for its chemical stability, durability, low cost, and nontoxicity (Nakata and Fujishima 2012). Alternative photocatalysts have been developed but many are impractical because of being based on expensive, rare, or toxic materials as well as fragility and chemical instability. Thus, TiO_2_ remains a popular choice (Loeb et al. 2019).

Studies have been performed on increasing the removal of organic pollutants by using hybrid photocatalytic technologies, such as a photocatalytic circulating-bed biofilm reactor with photocatalytic-biological carriers (Marsolek et al. 2008), photocatalytic membrane reactor consisting of a photocatalyst deposited on ceramic membranes (Lim and Goei 2016), and sonophotocatalysis (the simultaneous application of photocatalysts and ultrasound (US)) (Kakavandi et al. 2019). In other studies, photocatalytic oxidation has been improved by using a US-generated mist (Itoh and Kojima 2019; Kato et al. 2023).

Photocatalysis offers advantages of nonselective degradation and mineralization of a wide range of organics, low chemical consumption because the photocatalyst can be reused, and the option to use solar irradiation. The disadvantages of photocatalysis are the need for efficient light irradiation of the photocatalyst, operational costs, energy input of artificial light sources, photocatalyst fouling, instability and safety concerns for some photocatalysts, photocatalyst recovery for slurry reactors, and equipment costs.

##### Sonolysis

Sonolysis involves using US to degrade organic pollutants in wastewater. Irradiation by US results in cavitation; that is, the formation, growth, and collapse of bubbles generating hot spots of approximately 5,000 and 500 atm and shock waves (Suslick 1990). Cavitation causes thermal dissociation and the formation of radicals (e.g., HO•, O•, H•, and HO_2_•) that react with organic pollutants (Atalay and Ersöz 2021). The sonolysis frequency is an important parameter that determines whether physical or chemical effects are dominant. Low US frequencies of 20–80 kHz are considered to mainly cause physical effects, whereas higher US frequencies of 150–2000 kHz are considered to mainly cause chemical effects (Chatel et al. 2017). The application of multiple frequencies may simultaneously enhance the cavitational intensity and chemical and physical effects (Gogate and Patil 2016). Sonolysis has advantages of safety, eco-friendliness, no addition of chemicals, utility as a pretreatment to enhance biodegradability, and the ability to degrade recalcitrant organics. The disadvantages of sonolysis are equipment costs, high energy consumption, and conversion of cavitational energy producing chemical and physical effects, which have limited the full-scale application of this technique (Pang et al. 2011; Pirsaheb et al. 2023). Sonolysis is versatile and compatible with other treatment techniques, such as biological treatment, photocatalysis, and the use of UV, O_3_, and H_2_O_2_ (Savun-Hekimoğlu 2020; Pirsaheb et al. 2023).

### Biological methods

Biological treatment uses microorganisms, such as bacteria, fungi, yeast, and algae, to remove pollutants from wastewater. Biological treatment of wastewater is mainly used to reduce organics but can also remove inorganic compounds, such as heavy metals (Singh et al. 2022). Biological treatment can effectively treat industrial wastewaters with high organic contents, such as those from the food, paper, and textile industries.

Biological treatment is typically categorized into aerobic and anaerobic types. Aerobic biological treatment consists of using microorganisms to convert organic pollutants into CO_2_, water, and biomass in the presence of oxygen, which is often supplied by mechanical aeration using air blowers and compressors. During anaerobic biological treatment, pollutants are metabolized by microorganisms in the absence of oxygen through anaerobic processes, including hydrolysis, acidogenesis, acetogenesis, and methanogenesis (Vijin Prabhu et al. 2021). As a result, biogas containing mainly CO_2_ and CH_4_ is produced (Vijin Prabhu et al. 2021). Aerobic treatment is usually used for low-strength effluents with CODs below 1000 mg/L, whereas anaerobic treatment is suitable for high-strength effluents with CODs above 4000 mg/L (Chan et al. 2009). Considering the greenhouse gas (GHG) emissions (CO_2_ and CH_4_), Cakir and Stenstrom (2005) reported that crossover points exist in the range of 300–700 mg/L ultimate BOD (BOD_u_), depending on the aerobic treatment efficiency. They indicate that anaerobic treatment emits less GHG for wastewater with a BOD_u_ value above the crossover point while aerobic treatment emits less GHG for a BOD_u_ value below the crossover point.

Anaerobic treatment offers advantages over anaerobic treatment, including a lower energy demand, six-to-eightfold lower biomass production, a smaller reactor volume, and the production of biogas, which can be used as fuel (Ghangrekar and Behera 2013). Ranieri et al. (2021) investigated the electricity consumption of 202 WWTPs in Italy and found an electricity consumption of 1.02 kWh/m^3^ for aerobic treatment and 0.43 kWh/m^3^ for anaerobic treatment. Aerobic treatment offers advantages over anaerobic treatment, including a higher quality of treated wastewater, reduced odor (Martin et al. 2011), and nutrient removal (Aziz et al. 2019). Thus, anaerobic–aerobic systems can be used to efficiently remove organic content and increase effluent quality to meet discharge standards (Chan et al. 2009). Other combined systems include AOAO, AAO, and AAOO. Biological treatment is combined with physical and chemical treatment, typically as a secondary treatment, in WWTPs.

#### Aerobic digestion

Commonly used aerobic treatment methods include AS, aerated lagoons, the sequential batch reactor (SBR), trickling filter, MBR, rotating biological contactor (RBC), and aerobic MBBR.

The handling of excess sludge has been a key issue in aerobic treatment. Although sludge is most commonly disposed of in landfills, other handling methods have been proposed because of the environmental impact of sludge, to comply with environmental regulations, and the increasing costs of landfill disposal (Nguyen et al. 2022). Some alternative methods for sludge disposal include sludge thickening followed by anaerobic digestion and incineration as well as dewatering and drying followed by incineration (Hao et al. 2020). The main disposal methods used in the EU have been sludge reuse (such as in agriculture) and sludge incineration (Kelessidis and Stasinakis 2012).

##### Activated sludge

Activated sludge is one of the most used biological processes for treating solids and organic pollutants in wastewater (Zhang 2020; Islam and Mahdi 2022). Microorganisms suspended in aeration tanks are used to remove organic pollutants and nutrients from wastewater. Following the aeration tank, sedimentation tanks are used to separate sludge, where some of the sludge is returned to the aeration tank to maintain the concentration of microorganisms, and the rest of the sludge is removed. Depending on the water quality demand or standards, the supernatant from the sedimentation tank may either be discharged directly or undergo further treatment or disinfection before discharge. Biocarriers may be used to increase the efficiency of COD removal by AS. Jagaba et al. (2022) reported up to 88.4% removal of COD in wastewater from the pulp and paper industry using a hydraulic retention time (HRT) of 2 days and rice-straw activated carbon. The advantages of AS include low installation costs, high effluent quality, and a low footprint. The disadvantages of AS include high operating costs, sludge disposal, and sensitivity to effluent characteristics (Rezai and Allahkarami 2021).

##### Sequential batch reactor

The SBR is a variation on AS in which unit operations, such as aeration and sedimentation, are carried out in the same tank (Albahnasawi et al. 2023). There are five stages of SBR operation. In the filling phase, wastewater is added to the tank. In the reaction phase, pollutants are removed with or without mixing and aeration. In the settling phase, the tank acts as a clarifier without inflow or outflow. In the drawing phase, the supernatant is discharged and excess sludge is removed. The idle phase is used to switch between tanks for multiple tank systems (Singh and Srivastava 2011). Aeration can be flexibly controlled in SBRs to realize aerobic or anaerobic conditions. The advantages of the SBR include flexibility, control, a small footprint, and low costs because unit operations can be conducted in a single tank. The SBR also has disadvantages, such as complex operation, maintenance, and possible sludge discharge and blockages in aeration equipment (U.S. EPA 1999).

##### Trickling filter

In trickling filters, wastewater is distributed over a bed of solid media, such as rocks, gravel, or plastic. The solid media provide a surface on which microorganisms can grow and form a biofilm. Aerobic conditions are achieved by either upward or downward natural airflow, depending on the temperature and the difference in the humidity inside and outside the trickling filter. Alternately, mechanical ventilation using low-pressure fans can be used to provide consistent upward or downward airflow (Daigger and Boltz 2011). Sloughing occurs as the microbial layer thickens, and a portion of the biofilm falls off into the effluent (U.S. EPA 2000). The sloughed film is separated out in a secondary clarifier featuring the secondary sludge. Trickling filters have been employed to treat wastewater from the dairy industry, achieving 85% COD removal using a HRT of 10 days at 7–13 °C (Shahriari and Shokouhi 2015). The SBR has been used to remove various pollutants in wastewaters from mining, textile, and other industries (Dhokpande et al. 2014). Trickling filters offer advantages such as simplicity, reliability, and low power requirements as well as disadvantages of odor emission and the need for additional treatment and operator monitoring (Rezai and Allahkarami 2021).

##### Membrane bioreactor

Membrane bioreactors combine biological treatment and membrane filtration (MF and UF). Biological treatment removes pollutants from effluent, and the generated sludge is separated by membranes rather than by sedimentation. There are external and submerged MBRs. Tubular membranes are installed separately from the bioreactor in external MBRs, whereas hollow-fiber or flat-sheet membranes are immersed in the bioreactor in submerged MBRs (Martínez et al. 2020). Aeration is used to provide air and turbulence, which is crucial for preventing membrane fouling in submerged MBRs (Melin et al. 2006). The use of ceramic filters with enhanced resistance to fouling and anaerobic operation has been proposed to reduce the energy demands of MBRs (Judd 2008). Some advantages of MBRs include the high effluent quality produced by membranes, small sizes, and simplicity of automation (U.S. EPA 2007). In an MBR, the HRT and solids retention time (SRT) can be controlled independently, enabling higher sludge concentrations, longer sludge retention times, and the development of specialized microorganisms (Melin et al. 2006). Membrane bioreactors have disadvantages such as fouling, the need for membrane maintenance, foaming, electricity demand up to double that of CAS, and high capital and operational costs (Al-Asheh et al. 2021).

##### Rotating biological contactor

Rotating biological contactors use rotating cylindrical discs that are partially submerged in wastewater (Waqas et al. 2023). Microorganisms consume organic matter and form a biofilm on the disk surface. The rotating discs promote oxygen transfer to maintain aerobic conditions and provide turbulence to remove excess solids from the disc (Cortez et al. 2008). Thus, the cost of aeration is lower than that for AS. The effluent from an RBC is sent to a secondary sedimentation tank to remove TSS (Pathan et al. 2016). The advantages of RBCs include small land usage, high biomass concentrations, low energy consumption, short HRTs, and low operational and maintenance costs. The disadvantages of RBCs include low flexibility, the need for sludge removal, and sensitivity to wastewater characteristics (Mizyed 2021).

##### Aerobic moving bed biofilm reactor

In MBBRs, suspended plastic biofilm carriers are used to support microbial growth and the development of biofilms. The plastic carriers have a similar density to that of water and are maintained in suspension by aeration, liquid circulation, or mechanical mixing (Gzar et al. 2021). Biofilm sloughing occurs in MBBRs, as in trickling filters. Biomass from the MBBR effluent is removed using methods such as sedimentation, flotation, microscreening, and membrane filtration (Ødegaard et al. 2010). A major advantage of MBBRs is the ability to upgrade and increase the performance of existing WWTPs that use AS, eliminating the need to build new tanks (Falletti et al. 2014). Upgrading AS to MBBRs can decrease the HRT, increase the SRT, prevent clogging and channeling, and reduce capital costs (Ahmadi et al. 2011).

##### Aerated lagoons

Aerated lagoons consist of basins or ponds in which mechanical aeration is provided by devices, such as floating surface aerators and submerged diffusers. Aerated lagoons may be further classified into complete- and partial-mix lagoons. Complete-mix lagoons are aerated to maintain solids in suspension, which requires a high energy input. The aerators in partial-mix lagoons are designed to provide oxygen and do not provide sufficient turbulence to maintain solids in suspension (Alvarado et al. 2013). Consequently, sludge settling occurs and anaerobic zones form. Partial-mix lagoons have one-tenth the energy demand of complete-mix lagoons (U.S. EPA 2002). The advantages of aerated lagoons include lower operation and management costs than those of AS, lower sludge generation than other secondary treatment techniques, and lower land usage than those of stabilization ponds. The disadvantages of aerated lagoons are larger land usage compared to that of AS, lower nutrient removal compared to that of stabilization ponds, and energy input for aeration (U.S. EPA 2002).

#### Anaerobic digestion

As reviewed by van Lier et al. (2015), the development of continuous anaerobic reactors began with low-rate anaerobic reactors like the single-flow through tank designed by Karl Imhoff in 1905 and continuous stirred tank reactors (CSTR) that were prevalent until the 1960s. These low-rate reactors have similar HRTs and SRTs, and due to the low growth rate of bacteria, they require large volumes to provide sufficient biomass concentration (van Lier 2008).

To increase the biomass concentration and enhance treatment capacity, high-rate anaerobic reactors were developed, which decoupled SRT and HRT. The first high-rate anaerobic reactors to be developed were the anaerobic contact process (ACP), which has a secondary clarifier and recirculates sludge similar to AS, and the anaerobic filter (AF), which uses support materials for microbial growth (van Lier et al. 2015).

In the 1970s, Lettinga et al. (1976, 1980) developed the UASB reactor. The expanded granular sludge bed (EGSB) reactor was developed to improve the UASB reactor so that higher loadings can be applied. High-rate anaerobic sludge bed reactors, including the UASB reactor, EGSB reactor, and their derivatives, have been the most popular anaerobic treatment for industrial wastewater, holding approximately 90% of the market share due to their compactness, simple operation, and ability to operate at high volumetric loading rates and short HRTs (van Lier 2008). Sludge retention in these reactors is increased by the effective separability and settleability of biomass.

The advantages of anaerobic reactors include efficient COD removal at high organic loadings, biogas production, low energy demand and operational costs, a small footprint, lower sludge production than AS, and generation of biologically stabilized sludge. The disadvantages of anaerobic reactors include the difficulty of controlling sludge granulation, the dependence of granulation on the wastewater quality, the need for pretreatment to remove TSS, sensitivity to shock loads (Hansen and Cheong 2007), temperature sensitivity, and long startup times.

The key conditions for anaerobic systems that can treat high COD loads include high sludge concentrations and retention, sufficient contact of biomass and wastewater, a high reaction rate, effective transfer of metabolic products from biofilms, adaptation of biomass to the wastewater characteristics, and favorable conditions for microorganisms (van Lier et al. 2020).

##### Upflow anaerobic sludge blanket

In UASB reactors, wastewater is fed from the bottom of the reactor and flows upward through a granular sludge bed and blanket. The biogas produced from the anaerobic digestion of organics provides mixing and facilitates the formation and maintenance of sludge granules (Daud et al. 2018). The formation of sludge granules is essential for UASB reactors because these granules support biofilms and provide buoyancy and settleability, facilitating the contact of biomass with wastewater and enabling biomass retention (Abbasi and Abbasi 2012). A gas–liquid–solid separator (GLSS), usually in the shape of a funnel or triangular prism, is installed in the upper section of the reactor to separate sludge granules, biogas, and water. The separated biogas and treated wastewater are discharged from the top of the reactor. The GLSS acts as a clarifier by separating and preventing mixing of the sludge granules and the effluent. The sludge concentration is maintained by the settling of the separated sludge granules. Efforts have been made to improve the GLSS design so that the sludge retention increases in the UASB reactor. Dos Santos et al. (2016) installed parallel plates at a 45° angle above the conventional separator, doubling the treatment capacity.

##### Expanded granular sludge bed

The EGSB reactor is a variant of the UASB reactor with a slight bed expansion resulting from a high superficial velocity of 4–10 m/h, which is achieved by using a tall reactor or effluent recirculation (Tauseef et al. 2013). An EGSB reactor has a specifically designed GLSS to separate biogas, effluent, and sludge. Compared to UASB reactors, EGSB reactors provide better contact between the biofilm and wastewater and may use higher sludge concentrations. Other advantages of EGSB reactors include a small footprint, the ability to function at high organic and hydraulic loadings, and the ability to treat wastewater containing lipids and toxic compounds (Mao et al. 2015). These advantages have resulted in an increasing number of installments of full-scale EGSB reactors compared to the declining use of UASB reactors (van Lier et al. 2020).

## Conclusions

A comprehensive overview has been provided of the characteristics of wastewater from various industries and wastewater treatment technologies, highlighting their principles, applications, advantages, and disadvantages. A wide variety of physical, biological, and chemical treatment methods exist to treat the diverse pollutants found in industrial wastewater. Each technology offers unique advantages in terms of efficiency, cost-effectiveness, selectivity, environmental compatibility, and resource recovery. Differences in wastewater characteristics, local site-specific factors, and treatment objectives make it necessary to implement wastewater treatment facilities tailored to these needs. A single technique often cannot adequately achieve the treatment objectives, motivating the combination of various techniques. However, only a few methods are used to treat industrial wastewater for technological and economic reasons (Crini and Lichtfouse 2019). Ongoing research and innovation in wastewater treatment continue to drive sustainable water usage. It is necessary to develop more advanced treatment methods to remove a wide range of pollutants in industrial wastewater to protect the environment, increase the quality of treated wastewater for recycling, reduce the resource intensity of wastewater treatment, and recover valuable resources. The complexity of wastewater management warrants the realization of technological advances and interdisciplinary collaboration to develop holistic approaches considering technological, economic, and social factors.

## Data Availability

The authors confirm that the data supporting the findings of this study are available within the article.
